# Ethnobotanical study of medicinal plants in the environs of Tara-gedam and Amba remnant forests of Libo Kemkem District, northwest Ethiopia

**DOI:** 10.1186/1746-4269-11-4

**Published:** 2015-01-07

**Authors:** Getnet Chekole, Zemede Asfaw, Ensermu Kelbessa

**Affiliations:** Department of Biology, Woldia University, P.O. Box 400, Woldia, Ethiopia; Department of Plant Biology and Biodiversity Management and The National Herbarium (ETH), Addis Ababa University, P.O. Box 3434, Addis Ababa, Ethiopia

**Keywords:** Ethiopia, Ethnobotany, Indigenous botanical knowledge, Medicinal plants, Tara-gedam

## Abstract

**Background:**

Remnant forests found in areas that have long been converted to agricultural landscapes are refuges of wild useful plants; and societies inhabiting them are custodians of rich indigenous botanical knowledge. This study was undertaken to document the medicinal plants used by the people living in and around Tara-gedam and Amba remnant forests, northwestern Ethiopia, together with the associated ethnomedicinal knowledge.

**Methods:**

Data were collected from 105 informants through semi-structured interviews, guided field walk, market survey; and analyzed using standard ethnobotanical analytical tools including ranking and comparison.

**Results:**

A total of 163 medicinal plant species in 145 genera and 67 families were recorded among which *Zehneria scabra* drew the highest community consensus. Seventy-one percent of the medicinal plants were those used for treating human ailments only, 21% for both human and livestock and 8% for livestock only. Asteraceae, with 14 species, had the highest number of medicinal plant species. The medicinal plants mainly (79.1%) belong to the shrub and herb categories and most of them were sourced from the wild habitats. Leaves and fresh plant materials were more frequently used for medicine preparation than other parts. Protected government and church forests as well as tree propagation in nurseries followed by planting them and local practices constitute the major forest conservation efforts that indirectly protect the medicinal plants in the area. Elders and healers knew more about the medicinal plants, their distribution, the local ethnomedicinal practices and knowledge transfer patterns. Though important for the local healthcare system and with potentials for modern drug discovery, both the plants and the knowledge pool are under threat.

**Conclusion:**

The diversity of medicinal plants and the associated indigenous knowledge of Tara-gedam and its environs are of a considerable value to the local community and beyond. There is, therefore, a need for conservation of the vegetation and the medicinal plants along with preservation of the wealth of the indigenous knowledge.

## Introduction

The relationship between plants and people is studied in ethnobotany, a field focusing on the study of the indigenous knowledge on how plants are perceived, used and managed [1, 2]. Indigenous knowledge refers to the knowledge, rules, standards, skills and mental sets generated by and kept in custody of local people in a particular area [3]. It is the result of many generations and long years of experiences, careful observations and trial and error experiments [4]; and this study focuses on the medicinal plants and the associated ethnomedicinal knowledge in the environs of Tara-gedam forest. The cultural and spiritual identity of indigenous peoples is often linked to intact primary forests with their rich biodiversity [5]. Hence, plant resources possess and preserve cultural heritages, biological information and indigenous knowledge on plant identity and utility [6]. The ethnobotanical literature [7] underlines that both saving plant species and documenting and preserving indigenous knowledge associated with them are fundamental urgent concerns. There are around 6,000 species of vascular plants in Ethiopia, out of which more than 14% are said to have been used as traditional plant medicines (TPMs) [8], while more than 1,000 species have been documented at the National Herbarium (ETH) database. Despite their treasured contributions, in particular in Ethiopia, thus far TPMs have been offered very little attention in modern research and development, while less effort has so far been made to upgrade the traditional herbal medical practices [9]. For the most part, the potential of practitioners of traditional herbal medicine to serve as partners in the process of drug discovery and in providing healthcare services is not equitably acknowledged [10]. Hence, documenting traditional medicinal plants and the related traditional medical knowledge is important in order to facilitate the discovery of new sources of drugs and promote sustainable use of natural resources in Ethiopia [11]. Tara-gedam forest, selected for the study, is among the national priority forest areas in Ethiopia [12] and Amba forest is found adjacent to it. Both these remnant forests are known as species rich forests in Amhara Region, and the nearby local communities are in constant interaction with the plant resources [13, 14], particularly so for those living in the forest fringes. Research revealed that urbanization in Ethiopia had tremendous impacts on the useful plants and the practice of traditional medicine [15]. Since Tara-gedam and Amba forests are found adjacent to the growing Addis Zemen Town, the impacts have already been alluded to by some researchers [14]. The local people, as in other parts of Ethiopia depend on traditional medicine, which mostly relies on medicinal plants, to fulfill their healthcare needs as pointed out by Zegeye [14]. Despite this fact, there are no studies on ethnomedicinal plants and the associated knowledge in the environs of Tara-gedam and Amba forests. Hence, this study was framed with the aim of documenting the medicinal plants and the associated ethnomedicinal knowledge of people living in the environs of Tara-gedam and Amba forests.

## Material and methods

### The study area and the demographic background

The study was conducted in the general environment of Tara-gedam and Amba forests, located in Libo Kemkem District (Wereda) in the South Gondar Zone of the Amhara Regional State, northwestern Ethiopia located at around 12°04.351′-12°10.926′N and 37°44.266′- 37°50.057′E. Tara-gedam forest ranges from 2062–2496 m a.s.l. and Amba from 2011–2541 m a.s.l. with the highest peak at Mt. Deboch. The climate data obtained from the National Meteorological Service Agency of Ethiopia shows that the mean annual maximum and minimum temperatures of the study area are 32.8°C and 8°C, respectively. The District receives a uni-modal rainfall of approximately 1300 mm per year and about 95.1% of the area is under moist weina dega (mid-highland) while the rest is under the wet Dega (highland) [16]. Medium and cold highland climatic features characterize the study area. The vegetation of the area belongs to the dry evergreen montane forest type consisting of forests, bushlands, shrublands and enrichment plantation interspersed with stands of natural vegetation [14]. Archival information [16] shows that forested land is about 4,429.5 hectares. Libo Kemkem District, in particular Tara-gedam, has several recreational sites. Mt. Kualla, along with diverse geographical features of the forest, Tara-gedam Monastery and many caves and forested churches are very useful for archaeological studies and for the tourism industry [17]. The 2007 census report of the Central Statistical Agency [18] of Ethiopia shows that Libo Kemkem District has an estimated population of 209,451 (106,564 males and 102,887 females). The inhabitants are mostly members of the Amhara ethnic community who speak the Amharic language with economies that are predominately based on rain-fed subsistence cultivation of crops mixed with livestock production [16]. There are 58 health services in the District [19]. Malaria, intestinal helminthiasis, and pneumonia were the top three human diseases and the major livestock ailments were pasteurllosis, anthrax, internal and external parasites, black leg, sheep pox, trypanosomiasis, respiratory tract infection, rabies and coccidiosis [20].

### Site selection methods and procedures

**Figure 1:**
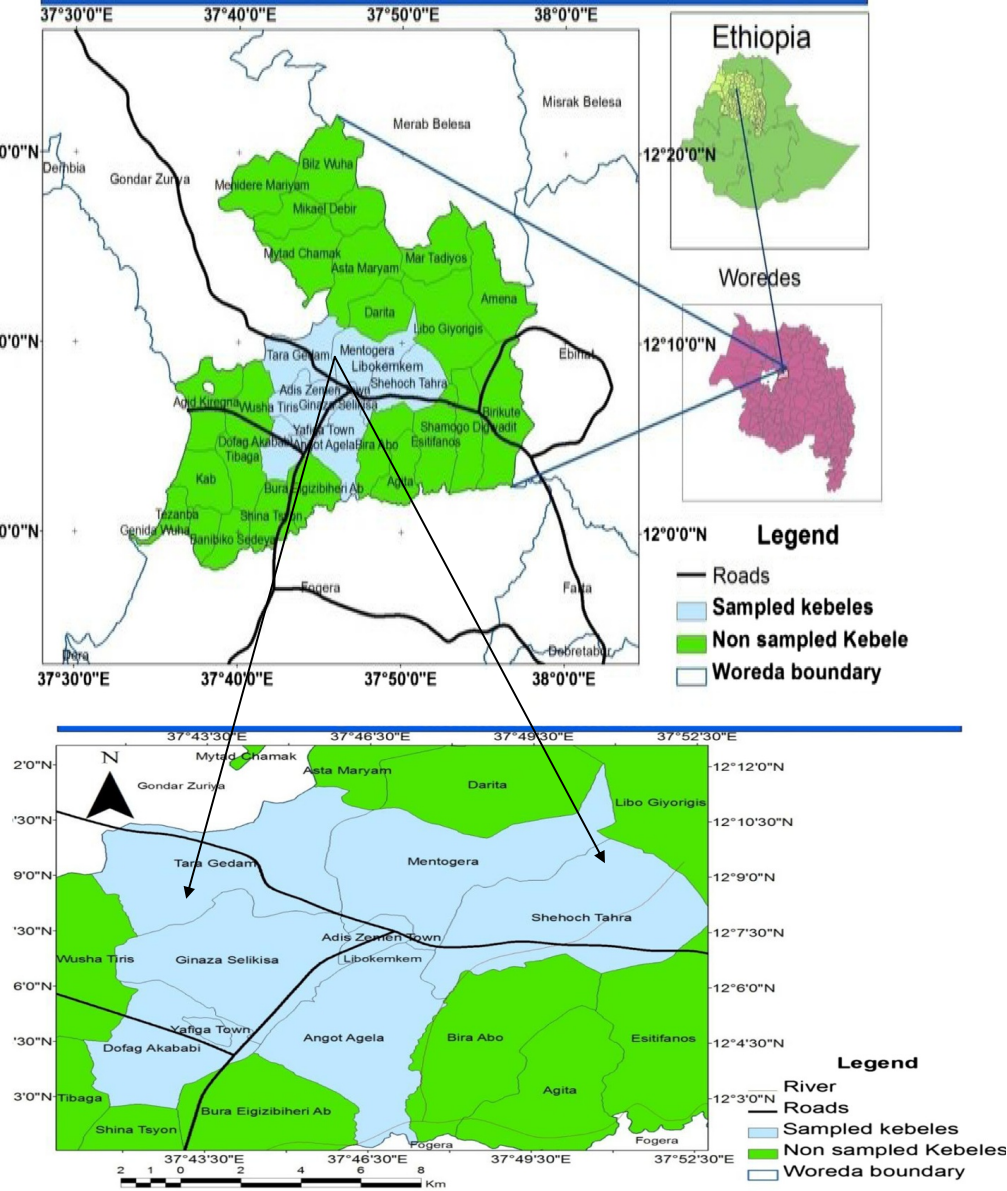
**Map of Ethiopia showing the regions,** **location of the study area and sampled kebeles.**

Before starting the ethnobotanical study, contacts were made with various offices (District administration, tourism and culture, agriculture and rural development, traditional healers’ association and health affairs) to seek permission to carry out the study by informing them about the aims and significance of the study. Letters authorizing the study were obtained from the relevant offices which were then presented to the concerned kebele (lowest administrative unit in Ethiopia) offices, forest scouts and informants in the study area. In this way, full legal procedures were followed and the informed consent of interested participants was obtained. Twelve rural villages, namely: Agamoch, Kidanemhret, Tibabosgie, Washa Indiras, Aguat Mafsesha, Mantogera, Abay, Kualla Yihuans, Yifag Akababi, Lomiye, Abuarra, Asiba Mariam and the town Addis Zemen were selected around the two forests. These villages are within the seven kebeles (Figure 1) selected for the study. Relative distance, community-forest interactions and altitudinal differences were the basic site selection criteria. Relative distance and community forest interaction were taken as criteria after collecting information from forest scouts, kebele administrative offices and inhabitants of the area during the reconnaissance survey in order to compare the indigenous knowledge of the communities found nearest to the forest with those found relatively far away (reached after traveling for more than five kilometers). This was undertaken from November-June 2010.

### Informant selection and approaches

One hundred five informants (85 males and 20 females) aged 19 to 84 were interviewed in this research. Among these, 45 (42 males and three females) were key informants and the rest 60 were general informants. Purposive and random sampling techniques were employed to select traditional herbalists and general informants respectively. The traditional association leaders, members of the tourism and culture office, elderly people and religious leaders helped to identify the key informants. In addition, the identified traditional practitioners and members who had earlier been treated by the healers also helped to identify other traditional experts. The general informants were randomly picked (from the list of inhabitants) during field and house visits (5–7 in each study site) by checking their names from the list of residents obtained from kebele offices. All interviews were administered after obtaining voluntary consent of each informant and assuring them that the data will be used only for academic purposes.

### Ways of data collection and type of data collected

Ethnobotanical data were collected during three months from November to January 2010 by living in close contact with the community in the study area, following standard methods [2, 4, 21]. Accordingly, semi-structured interview, guided field walk, direct observation, market survey and focus group discussions with key informants and other knowledgeable community members were applied and their knowledge on medicinal plants gathered.

Interviews were held based on checklist of questions prepared before hand in English language and simultaneously translated into Amharic. Interviews focused to informant’s demographic features including sex, age, marital status, occupation, religion, educational background, and duration of time an informant lived in the study area, and indigenous ecological knowledge (traditional ways of classifying vegetation, plants, landscapes and the soils in the area). The major part of the interviews were focused on the local names of medicinal plants used, their habits and habitats, plant part/s used, remedy preparation methods, materials used during preparation, condition of preparation, storage method, additives/ingredients used during preparation and administration, dosages administered, and route of administration. Likewise, side effect of the medicine (if any), use of antidotes for adverse effects, any taboos associated with medicinal plants, the season, month, dates and time of collection and preparation of plant medicines, and market value were also included. Further, the distribution (status) of medicinal plants, the interaction of healers with the District administration, threats and major problems, conservation methods, source of knowledge and ways of transfer and number of years of service as traditional healer were also the major interview points targeted, following the methods used by previous investigators [2, 4, 22].

The semi-structured interviews held with informants usually started at their sitting places and further broadened into field walk with interviewed informants in order to see the plants mentioned in their habitats and voucher collections following Martin [4]. This activity further helped to record growth habits of medicinal plants. Focus group discussions were done with traditional medicinal plant association members, other herbalists, monks and general informants to obtain additional information and to check the reliability. Informants were contacted two to three times and responses of an informant in harmony with each other were taken as relevant and used for data analysis. At times, the preparation methods of the medicinal plants were said to be secret and were not included during discussion. Most field observations were conducted with a single informant in order to keep the knowledge top-secret as this was what the healers in particular preferred. Some of the traditional healers were genuine herbalists, well-known by the local community and owned traditional home pharmacies derived from plant remedies. They were asked to demonstrate their work at their homes and in the field, which was recorded in order to check the consistency in knowledge and practice on the preparation of remedies and their effectiveness. The patients encountered at healers’ homes were also asked about the traditional plant medicines they have used and their effectiveness when applied by healers.

### Plant collection and identification

Voucher specimens were collected for each plant species during guided field walk with the informants. At times, the field activities included taking notes on plants and the associated indigenous knowledge with preliminary identification of the plants to family and sometimes to species levels. Photographic records were also taken in the field to capture the field sites, plants and other useful memories. The specimens were dried, deep-frozen, and determinations were made at the National Herbarium (ETH), Addis Ababa University, using taxonomic keys and descriptions given in the relevant volumes of the Flora of Ethiopia and Eritrea [23–31] and by visual comparison with authenticated herbarium specimens. Finally, the accuracy of identifications was confirmed by a senior plant taxonomist and the voucher specimens with labels were deposited at the ETH.

### Data analysis

The ethnobotanical data were analyzed using Microsoft Office Excel spreadsheet (2007) and SPSS version 20 software. The former was used to calculate sum, percentages, tabulate and draw graphs whereas the latter was used to generate results of descriptive statistics, and perform t-tests as well as draw graphs and charts. Ethnobotanical ranking and scoring methods such as preference and direct matrix rankings as well as pair-wise comparisons and informant consensuses were employed to distinguish priority species and to check consistency.

Preference/priority ranking activities were employed on six most preferred and widely used medicinal plant species for the treatment of wound and the most threatened medicinal plants. Direct matrix ranking was employed for the six most widely utilized multi-purpose plant species and for the five factors considered most threatening to medicinal plants. Pair-wise comparison was made on six of the most preferred and commonly used medicinal plants against stomachache. To do this, the number of possible pairs was determined by applying the formula n(n-1)/2, where n is the number of medicinal plant species being compared. For all the above ethnobotanical ranking and scoring techniques, the same seven key informants who had long time practical experience in traditional plant medicine preparation, administration and collection were engaged. The strength of knowledge of the key informants was evident to the first author who witnessed the clarity of explanations and accuracy of actions. The overall procedures for these activities were conducted following standard ethnobotany texts [2, 4, 22]. Informant consensus factor (ICF) for different ailment categories was calculated to test agreements of the informants on medicinal plant knowledge of each category by using the formula ICF = Nur-Nu/Nur-1 where, nur is the number of uses reported in each category and Nu is the number of species reported in each category [32].

### Ethical consideration

All data collections were done with special care on the base of the cultural view of the local communities in the study area. Informants were also informed that the objectives of the research were not for commercial purposes but for academic reasons. Since, ethnomedicinal indigenous knowledge is only obtained from traditional specialists within the community so any value that will obtain as a result of the research will benefit the community. According to ethnobiology code of ethics indigenous knowledge should be protected and a part of the value generated should be transferred back to the authors of the knowledge. Finally, informants were accepted the idea and came to reach an agreement.

## Results

### Demographic features of the informants

Of the total informants, 46 were in the age group of 51–85; 51 were illiterate and the greater proportion (88) belonged to the married category. Almost all informants (101) belonged to the Ethiopian Orthodox Church. Parallel to the population structure, there were more males than females who were willing to be included among informants as indicated by the demographic profile in Table 1.

**Table 1:** **Demographic profile of the informants**

Sex	Age group (in yrs)			Educational status			Marital status		Religious type	
	19-34	35-50	51-85	Illiterate	Religious education	Modern education	Single	Married	Orthodox	Muslim
Male	22	25	38	38	17	30	9	76	83	3
Female	5	7	8	13	0	7	8	12	19	1
**Total**	27	32	46	51	17	37	17	88	101	4

Most informants (70) were farmers, 11 of them were house wives, seven were students and other groups were represented by fewer numbers. Of the total informants, 99 lived in the study area since birth and the rest have lived there from six to 20 years.

### Indigenous ecological knowledge of people in the study area

The inhabitants of the study area are owners of rich ethnobotanical and ethnoecological knowledge as demonstrated by their wide array of knowledge on environmental matters. They classified the land forms; vegetation and soil based on knowledge surviving from ancestral practices (Table 2), now evident through their elaborate emic categorization systems.

**Table 2:** **Emic categorization of landscape**, **soil and vegetation in the area**

Landscape(ethno-topographic)types		Soil (ethnopedologic and scientific) types			Vegetation (ethnofloristic) types	
Amharic	English	Amharic	English	Scientific	Amharic	English
WOTAGEBA	Up and down	KEYATIE	Red soil	Luvisols	KUTQUATO	Shrub
TERRARAMA	Mountainous	WALKA	Black soil	Vertisols	GITOSH	Grass land
MEDAMA/MESK	Plain	SERBOLA	Black & white	Anthrosols	CHAKA	Forest
SHELEQUAMA	Valley	CHINCHA	Brown	Leptosols	DENE	Plantation
KOREBTA/GOBA	Outcrop land	BORENK	White	Cambisols	CHEBECHEB	Wetland (edaphic grassland)
DAGET	Hilly					
SINKURKUR	Stony place					

### Medicinal plant diversity and distribution

The study documented 163 species of medicinal plants belonging to 145 genera and 67 families. Three of the families had ten or more species each and the details are given in Table 3 and Table 4. The medicinal plant use reports showed that six species were cited by more than 20 informants each (Table 5). Twelve species were cited for the treatment of six and more ailments each (Table 6). *Achyranthes aspera* came out on the lists of both most effective and most cited medicinal plants and the details are tabulated (Table 4 and Table 5).

**Table 3:** **Plant families**, **number of medicinal plant species and proportions**

No	Family	No of species in each	% of total
1	Asteraceae	14	8.6
2	Fabaceae	13	8.0
3	Solanaceae	10	6.1
4	Euphorbiaceae	8	4.9
5	Lamiaceae	7	4.3
6	Malvaceae	6	3.7
7	Apiaceae	5	3.1
8	Acanthaceae, Amarantaceae, Asclepiadaceae, Cucurbitaceae, Rubiaceae, Rutaceae (six families)	4	2.5
9	Convolvulaceae, Moraceae, Rhamnaceae, Poaceae, Polygonaceae, Oleaceae (six families)	3	1.8
10	Boraginaceae, Cuppressaceae, Loganiaceae, Myrsinaceae, Myrtaceae, Ranunculaceae, Rosaceae, Scrophularaceae, Urticaceae, Apocynaceae (ten families)	2	1.2
11	Other 38 families	1	0.6

**Table 4:** **List of plant species used to treat human and livestock ailments: scientific names, family, vernacular name, growth forms (Gf), Ailments treated, Ailment type(At), parts used (Pu), condition of preparation (Cp), route of administration (Ra), method of preparation, habitat (Ha), distribution(Dn), collection number (Co.No.) in the environ of Tara-gedam and Amba forests**

Scientific Names	Family	Vernacular name (Amharic)	Gf	Ailments treated	At	Pu	CP	Ra	Methods of preparation	Ha	Dn	Co.No.
*Acacia abyssinica* Hochst. ex Benth.*	Fabaceae	Girar	T	Scorpion poison	Hu	B	F	De	Tie with inside part	F	Spr	GC097
*Acanthus polystachius* Del*.*	Acanthaceae	Nech kusheshile	S	Rabies	Li	R	F	O	Pound and give with water	Fal	Spr	GC031
*Acanthus sennii* Chiov.*	Acanthaceae	Key kusheshilie	S	Evil eye	Hu	R	FD	Na, O & De	Sniff, drink and fumigate with concoction	F	Wy	GC056
				Arthritis/rheumatism	Hu	R	F	De & O	Crush & tie or drink with honey	Bo		
				Tape worm	Hu	R	F	O	Pound, immerse in water then drink the juice			
*Achyranthes aspera* L.	Amaranthaceae	Telenj	H	Eye problem	Hu	L	F	Op	Pound, immerse to water, squeeze and insert with cotton		Wy	GC025
				Wound	Hu	L	FD	De	Crush, powder and tie			
				Wound	Hu	L	F	De	Crush and tie			
				Excessive menstruation	Hu	R	F	O	Crush, insert in water then drink juice			
				Tonsillitis	Hu	L	F	De	Crush and tie			
				Bleeding	Li	R	F	De	Crush and tie			
				Bone fracture	B	R	FD	De	Tie the concoction			
				Bleeding	Hu	R	FD	De	Tie the concoction			
				Tape worm	Hu	R	F	O	Crush, insert in water then drink			
*Acmella caulirhiza* Del.	Asteraceae	Kutchamelk	H	Swelling	Hu	L	FD	De	Crush and powder then tie with honey/better	Hg	Pa	GC134
*Acokanthera schimperi* (A.DC.) Schweinf.	Apocynaceae	Merz/Mirez	S	Spider poison	Hu	L	D	De	Crush and powder then cream with butter	Bo	Rr	GC047
				Hepatitis	Hu	Ap	D	Na, O & De	Crush, dry then fumigate			
*Adiantum capillus-veneris* L.	Adiantaceae	Joroasfit	H	Anthrax	Hu	R	F	O	Crush, insert in water then drink the juice	F	Spr	GC027
				Ear wound	Hu	St	FD	De	Insert into new jewelry hole			
*Allium sativum* L.	Alliaceae	Nech shinkurt	H	Evil eye	Hu	Bu	F	Na, O & De	Sniff, drink and fumigate with concoction	Hg	Spr	GC011
				Malaria	Hu	Bu	F	O	Crush and drink with honey or smash in water then drink			
				Influenza virus	Hu	Bu	F	O	Crush and drink with water			
				Febrile illness	Hu	Bu	F	O	Crush then fumigate or drink the concoction			
				Pneumonia	Hu	Bu	F	O	Chop and eat with honey			
*Aloe macrocarpa* Tod.	Aloaceae	Eret	H	Impotency	Hu	R	F	De	Crush and powder, then cream with butter	Fwl	Rr	GC034
				Wound	B	Lx	F	De	Creamed			
*Alternanthera pungens* Kunth	Amaranthaceae	Midir akef	H	Babies diseases	Hu	L	F	De	Rub, squeeze then cream	Bo	Rr	GC146
*Alysicarpus quartinianus* A.Rich.	Fabaceae	-----------	H	Ascaris	Hu	R	F	O	Crush then drink with milk	Fwl	Rr	GC142
*Argemone mexicana* L.	Papaveraceae	Yahya eshoh	H	Rabies	Li	R	F	O	Crush then give with water	Rs	Wy	GC058
*Artemisia afra* Jack. ex Willd.	Asteraceae	Chikugn	H	Evil eye	Hu	Ap	FD	Na, O & De	Sniff unprocessed and powder then fumigate and drink concoction	Hg	Rr	GC168
*Asparagus africanus* Lam.	Asparagaceae	Yesiet kest	S	Impotency, gonnoria,& syphilis	Hu	R	DF	O	Crush, infusion with honey then drink the juice	Fal	Spr	GC151
				Itchiness	Hu	R	DF	De	Crush, powder then cream with butter			
				Excessive menstruation	Hu	R	F	O	Chew and swallow the juice			
				Evil eye	Hu	R	DF	Na, O & De	Sniff, drink and fumigate concoction			
*Astragalus atropilosus* (Hochst.) Bunge	Fabaceae	-----------	H	Itchiness	Hu	Ap	D	De	Dry, burn then cream ash with butter	Fal	Spr	GC152
*Bersama abyssinica* Fresen.	Melianthaceae	Azamir	S	Ascaris	Hu	L	FD	O	Crush and powder, boil with tea then drink juice	Aw	Spr	GC107
*Bidens macroptera* (Sch Bip.) ex Chiov. Mesfin	Asteraceae	Adey Abeba	H	Brain cancer	Hu	Fl	D	Na	Powdered	Fal	Wy	GC143
*Brassica carinata* A. Br.	Brassicaceae	Gomen	H	Stomachache & Anthrax	B	Sd	D	O	Grind and drink with water	Hg	Wy	GC176
*Bridelia micrantha* (Hochst.) Brain.	Euphorbiaceae	Yenebr tifir	T	Expel placenta	Li	B	F	O	Crush then give with water	Rs	Rare	GC089
*Brucea antidysenterica* Swiss Chard.	Simaroubaceae	Waynos/yedaga abalo	H	Wound & Scabies	Hu	L	D	De	Crush, mixed with butter then cream	Fal	Spr	GC086
				Skin rash	Li	L	D	De	Crush, mix with butter then cream			
*Buddleja polystachya* Fresen.	Loganiaceae	Anfar	S	Tonsillitis	Hu	Sh	F	De	Tie and cream concoction	F	Spr	GC062
				Intestinal parasite	Hu	L	D	O	Crush and powder, immerse in tej then drink the juice			
				Excessive menstruation	Hu	L	F	Va	Make soft by rubbing, and insert with new cloth until bleeding stops			
				Wound	Hu	Sh	F	De	Crush and tie			
*Calotropis procera (*Ait.) Ait.f.	Asclepiadaceae	Tobia	S	Hemorrhoid	Hu	Lx	F	De	Cream concoction	Rs	Spr	GC035
				Expel spine in wound	Hu	Lx	F	De	Cream on point			
*Calpurnia aurea (*Ait.) Benth.	Fabaceae	Zikita	S	External parasites	Li	L	F	De	Crush, then wash with water	Bo	Spr	GC020
				Diarrhea & Bilharziasis	Hu	Sd	D	O	Grind and eat after pounding with honey			
				Bloody diarrhea	B	R	F	O	Crush then drink with water			
				Erthroblastosis	Hu	Sd	D	De & O	Grind and drink with honey or tie powder/concoction on neck			
				Expel foreign things from eye	Hu	L	F	Et	Crush mixture, squeeze then insert with cotton wool			
				Prolonged embryo in uterus	Hu	R	DF	De	Tie concoction on spinal column			
*Capparis tomentosa* Lam.*	Capparidaceae	Gimero	S	Evil eye	Hu	R	DF	Na, O & De	Sniff, drink and fumigate concoction	F	Wy	GC023
				Epidemic	Hu	R	D	De	Burn the concoction and fumigate			
*Capsicum annuum* L.	Solanaceae	Karia/keto	H	Malaria	Hu	Fr	F	O	Crush and drink with honey or smash in water then drink	Hg	Wy	GC026
*Carica papaya* L.	Caricaceae	Papya	T	Malaria	Hu	L	F	O	Crush and drink with milk	Hg	Spr	GC098
				Cough	Hu	R	F	O	Crush and boil with tea then drink juice			
*Carissa spinarum* L. *	Apocynaceae	Agam	S	Evil eye	Hu	R	FDD	Na, O & De	Sniff, drink and fumigate concoction	F	Wy	GC021
				Epidemic	Hu	R	D	Na, O & De	Burn the mixture and fumigate			
				Brain tension/stress	Hu	R	D	Na	Crush then fumigate			
*Cayratia gracilis* (Guill.&Perr.) Suesseng	Vitaceae	Aserkush	Cl	Hemorrhoid	Hu	R	F	De	Cream concoction	Fwl	Spr	GC052
*Celosia trigyna* L.	Amaranthaceae	Lemlemcho	H	Tape worm	Hu	Sd	D	O	Grind and drink with water	Hg	Spr	GC132
*Chenopodium murale* L.	Chenopodiaceae	Amedmado	H	Wound	Hu	L	DF	De	Crush then cream with butter	Hg	Rr	GC136
				Ear problem	Hu	L	F	De	Concoction inserted to ear tube			
*Cicer arietinum* L.	Fabaceae	Shinbira	H	Malaria	Hu	Sd	D	O	Germinate then eat with bulb of *Allium sativum*	Bo	Wy	GC115
*Cirsium englerianum* O. Hoffm.	Asteraceae	Yahyakusheshilie	H	Beating with stick	Li	St	F	O	Crush, immerse in water then drink juice	F	Spr	GC050
				Scabies	Hu	Sh	F	De	Crush, roast then cream			
				Influenza virus	Hu	Fr	F	O	Crush and drink with water			
*Citrus aurantifolia* Burn. f.	Rutaceae	Lomy	S	Wound	Hu	Fr	F	De	Cream concoction	Hg	Spr	GC169
*Citrus aurantium* L.	Rutaceae	komtatie	S	Hypertension	Hu	Fl	F	O	Drink the juice	Hg	Rr	GC138
*Clausena anisata* (Willd.) Benth.	Rutaceae	Limich	S	Evil eye	Hu	R	D	Na, O & De	Sniff, drink and fumigate with concoction	F	Spr	GC178
*Clematis simensis* Fresen.	Ranunculaceae	Azo areg	Cl	Hemorrhoid	Hu	L	F	De	Crush then tied	F	Spr	GC043
				Wound	B	L	F	De	Crush then tied			
				Cancer	Hu	L	F	De	Crush and powder then cream			
*Clerodendrum myricoides* (Hochst.) Vatke	Lamiaceae	Misroch	S	Evil eye & evil sprit	Hu	L,R &Sd	FD	De & O	Crush, powder then tie on the neck or take with tooth	F	Spr	GC016
*Clutia lanceolata* Forssk.	Euphorbiaceae	Fiyelefej	S	Diarrhea	Hu	R	F	De	Crush then tie on neck region	Fwl	Wy	GC135
				Bone fracture	Hu	R	F	De	Crush and tie			
				Beating with stick	Li	L	F	O	Crush and give with water			
				Expel ear mites	Hu	Fr	F	Et	Grind, insert into ear tube until it expels mites			
*Coffea arabica* L.	Rubiaceae	Bunna	S	Common cold	Hu	L	F	O	Boil, decant then drink the juice	Hg	Spr	GC161
				Diarrhea	Hu	Fr	F	O	Grind and eat with honey			
*Commelina latifolia* Hochst. ex A Rich.	Commelinaceae	Yewuha enkur	H	Wound	Hu	L	F	De	Crush and tie	Ris	Spr	GC116
				Taenia scaplis	Hu	L	D	De	Crush and powder then cream with butter			
*Convolvulus arvensis* L.	Convolvulaceae	Este filastot	H	Impotency	Hu	R	DF	O	Crush and powder then drink with GIN (areki)	Fwl	Rr	GC175
				Anthrax	Hu	R	F	O	Peel, chew then swallow juice			
*Convolvulus sagittatus* Thunb.	Convolvulaceae	--------------	H	Anthrax	Hu	R	F	O	Peel, chew then swallow juice	Ah	Rr	GC127
*Cordia africana* Lam.*	Boraginaceae	Wanza	T	Eye problem	Li	L	DF	Op	Burn, then insert ash with butter	Bo	Wy	GC133
				Fire burn	B	L	DF	De	Burn, then cream the ash			
				Anthrax	Li	L	F	O	Crush and give with water			
				Expel ear mites	Hu	L	F	Et	Rub, squeeze, insert then cover cotton			
*Crepis rueppellii* Scli-Bip.	Asteraceae	-----------	H	Anthrax	Li	R	F	O	Crush and give with water	Fwl	Rr	GC070
*Crotalaria karagwensis* Taub.	Fabaceae	Yeayt ater	H	Itchiness	Hu	L	FD	De	Crush and powder then cream with butter	Ah	Rr	GC051
*Croton macrostachyus* Del.	Euphorbiaceae	Misana	T	Intestinal & abdominal problems	Hu	L	F	O	Boil, grind then eat with butter, shirro or teff injera	Aw	Wy	GC130
				Stomachache	Hu	Sh	F	O	Drink concoction			
				Bloating	Li	Sh	F	O	Crush and give with water			
				Ring worm	Hu	Sp	F	De	Cream affected part			
				Evil eye	Hu	R	DF	De & O	Sniff and drink the concoction			
				Snake poison	Hu	R	F	O	Crush and drink with water			
				Tape worm	Hu	B	F	O	Crush, pound, then drink juice			
				Tape worm	Hu	L	F	O	Boil, grind, make it WOTE (souse) with butter then eat with ENJERA			
				Paralyzed leg	Hu	R	DF	De	Crush with *Carissa spinarum* root mix with water and immerse affected part			
*Cucumis ficifolius* A. Rich.	Cucurbitaceae	Yemidir enbuay	H	Bloody diarrhea	B	R	F	O	Crush and mix with milk	Bo	Rr	GC139
				Evil eye	Hu	R	DF	Na, O & De	Sniff, drink and fumigate concoction			
				Stomachache & Anthrax	Hu	R	F	O	Peel, chew then swallow juice or crush and drink with water			
				Evil eye	Hu	R &Fr	FD	De & O	Crush and tie on neck			
				Wound	Hu	Fr	F	De	Insert the affect part into the fruit			
				Expel ear-mites	Hu	Sh	F	Et	Crush, squeeze then insert			
*Cucurbita pepo* L.	Cucurbitaceae	Duba	Cl	Expel placenta	B	Fr	F	O	Chop then boil with water	Hg	Spr	GC166
				Heart & gastritis problems	B	Fr	F	O	Chop then boil with water			
				Sterile females	Hu	R	F	O	Chew and swallow juice to be fertile	F	Wy	GC082
*Cupressus lusitanica* Mill.	Cuppressaceae	Yeferenge tid	T	Tooth ach	Hu	L	F	O	Boil with salt then take with teeth			
*Cyathula prostrata* (L.) Brume	Amaranthaceae	Aregist	H	Anthrax	Li	L	F	O	Rub, squeeze then give with water	Hg	Pa	GC145
*Cynodon dactylon* (L.)Pers.*	Poaceae	Serdo	H	Snake poison	Hu	Ag	F	O	Chew and absorb the juice	Bo	Wy	GC173
				Tape worm	Hu	L& St	F	O	Drink the concoction			
*Cynoglossum coeruleum* (Hochst. ex A.Rich.) DC	Boraginaceae	Chegogit	H	Febrile illness	Hu	L	F	De & O	Rub, squeeze then cream and drink the juice	Bo	Wy	GC114
				Expel foreign things from eye	Hu	L	F	Op	Crush mixture, squeeze then insert with cotton wool			
				Spider poison	Hu	L	F	De	Crush, pound then cream with butter			
				Wound	Hu	L	F	De	Crush then cream			
				Eye problem	Hu	L	F	Op	Rub, squeeze then insert one-two droplets			
				Expel ear-mites	Hu	L	F	Et	Rub, insert and squeeze			
*Cyperus dichroostathyus* A.Rich.	Cyperaceae	Giramta	H	Trachoma	Hu	Fl	FD	Op	Burn and cream the ash with butter	F	Wy	GC113
*Datura stramonium* L.	Solanaceae	Astenagir	H	Scabies and ear wound	Hu	L	F	De	Crush then cream	Bo	Wy	GC124
				Expel foreign things from eye	Hu	L	F	Op	Crush mixture, squeeze then insert with cotton wool			
*Dichondra repens* J.R.&G.Forst.	Convolvulaceae	Afer kocher	H	Febrile illness	Hu	L	F	De	Rub, squeeze then cream except heart	Fwl	Rr	GC180
*Diplolophium africanum* Turcz.	Apiaceae	Zegerawta	H	Headache	Hu	L	F	Na	Sniff the unprocessed leaf	F	Rr	GC041
				Rabies	Li	R	F	O	Pound and give with water			
*Dipsacus pinnatifidus* Steud. ex A. Rich.	Dipsacaceae	Ferezeng/kelem	H	Rabies	Hu	L	F	Na	Pound and give with water	F	Spr	GC102
*Discopodium penninervium* Hochst.	Solanaceae	Almit	S	Beating with stick	Hu	Sh	F	Na & Et	Crush and give with water	Fal	Rr	GC071
*Dodonaea angustifolia* L.f.	Sapindaceae	Kitkita	S	Scabies	Hu	L	F	De	Crush and cream with butter	F	Wy	GC036
				Bone fracture	Li	L& St	F	De	Tie twig parts together			
				Tape worm	Hu	R & L	F	O	Pound, immerse in water and drink the diluted mixture			
				Tape worm	Hu	L& St	F	O	Drink the concoction			
*Dovyalis abyssinica* (A. Rich.) Warb.*	Flacourtiaceae	Koshim	S	Hemorrhoid	Hu	Fr	F	De	Immerse in water in flat material and sit on	Bo	Rr	GC042
*Dregea rubicunda* Schum.	Asclepiadaceae	Kuandira	Cl	Rabies	Hu	L	F	O	Crush and drink with milk	F	Rr	GC044
				Wound	Hu	L& B	D	De	Crush, powder then tie			
*Dyschoriste radicans* Nees	Acanthaceae	----------------	H	Stomachache	Hu	Ap	F	O	Chew and swallow the juice	Fwl	Rr	GC093
*Embelia schimperi* Vatke*	Myrsinaceae	Enkoko	S	Tape worm	Hu	Fr	FD	O	Eat fresh or crush and drink with 'tela difdif’	Ris	Rr	GC119
*Eragrostis tef* (Zucc.) Trotter	Poaceae	Tef	H	Dandruff	Hu	Sd	D	De	Grind, prepare dough then cream on bare head	Hg	Wy	GC040
				Bloating	Li	Sw	D	O	Give the straw			
*Erythrina abyssinica* Lam. ex DC.	Fabaceae	Kuara	T	Febrile illness	Li	B	D	De & O	Crush then fumigate or drink the concoction	Ah	Rr	GC159
*Eucalyptus globulus* Labill.	Myrtaceae	Nech bahirzaf	T	Febrile illness & Common cold	Hu	L	F	Na, O & De	Boil and fumigate with the fume	Fal	Rr	GC167
*Euclea racemosa* Hiern	Ebenaceae	Dedeho	S	Scorpion poison	Hu	R	F	De	Crush and tie	F	Spr	GC018
				Gonorrhea	Hu	R	FD	O	Boil, crush then eat with honey or butter			
				Eye problem	Li	R	F	Op	Peel and cream with butter for one night then use butter for paint			
				Toothache	Hu	Rb	F	O	Take with teeth			
				Prolonged embryo	Hu	R	DF	De	Tie the concoction on spinal column			
*Euphorbia abyssinica* Gmel.	Euphorbiaceae	Kulkual	T	Jaundice	Hu	R	F	O	Crush, immerse in water then drink or bake with bread then eat	Bo	Wy	GC164
				Stomach and intestinal problems	Hu	R	F	O	Crush, mix with DORO WOTTE then eat with ENJERA			
				Rabies	Li	Lx	F	O	Mix with milk			
				Malaria	Hu	Lx	F	O	Eat bake with *Eragrostis tef* dough			
				Hemorrhoid	Hu	Lx	F	De	Cream the concoction			
				Skin diseases	Hu	Fl	D	De	Crush, powder, then cream with honey			
*Euphorbia tirucalli* L*.*	Euphorbiaceae	Kinchib	S	Wound	B	Lx	F	De	Paint the affected part	Ah	Wy	GC131
				Hemorrhoid	Hu	Lx	F	De	Cream the concoction			
				Wound	Hu	Lx	F	De	Cream the concoction			
*Ferula communis* L. *	Apiaceae	Dog	H	Increase sexual needs	Li	R	F	O	Pound, then give with INGERA and butter	F	Wy	GC072
				Evil sprit	Hu	R	DF	De	Crush and fumigate			
				Blood flow	Hu	R	F	De & O	Crush, immerse in water then give for newly delivered mother			
				Lung cancer (TV)	Hu	R	F	O	Crush and drink with water			
				Erthroblastosis	Hu	R	DF	De	Grind and drink with honey or tie powder (concoction) on neck			
				Impotency	Hu	R	F	O	Drink concoction with honey			
*Ficus carica* L.	Moraceae	Beles	S	Wound	Hu	Lx	F	De	Cream the affected part	Fwl	Rr	GC104
*Ficus sur* Forssk.*	Moraceae	Sholla	T	Toothache	Hu	B	F D	O	Take by teeth	Ris	Spr	GC090
*Ficus vasta* Forssk.*	Moraceae	Warka	T	Wound	Hu	Lx	F	De	Cream the concoction	Fal	Rr	GC162
*Foeniculum vulgare* Miller	Apiaceae	Ensilal	H	Cough	Hu	Ag	F	O	Boil with tea then drink	Bo	Rr	GC137
				Asma		L& St	F	O	Crush, immerse with milk then drink			
				Urinary retention	Hu	L& St	F	O	Cook in water then drink the juice			
*Gardenia ternifolia* Schumach. & Thonn.*	Rubiaceae	Gambillo	T	Erthroblastosis	Hu	R	DF	De	Grind and drink with honey or tie powder/concoction on neck	Bo	Rr	GC087
*Gossypium barbadense* L.	Malvaceae	Tit	S	Snake bite	Hu	R	DF	De & O	Tie on neck or chew, absorb the juice	Hg	Rr	GC096
				Tonsillitis	Hu	Fr	D	O	Grind then drink the liquid			
*Grewia ferruginea* Hochst. ex A. Rich.*	Tiliaceae	Lenquata	S	Expel placenta	Li	B	F	O	Peel the inside part, chop, emulsify with water then give	F	Wy	GC123
				Dandruff	Hu	B	F	De	Wash with inside part			
*Guizotia schimperi* Sch. Bip.ex Walp.	Asteraceae	Mech	H	Stomachache	Hu	R	F	O	Chew and swallow the juice	Fwl	Wy	GC073
				Wound	Li	Ag	F	De	Rub the part affected by ticks			
*Helinus mystacinus* (Ait.) E. Mey. ex Steud.	Rhamnaceae	Esat abrid	Cl	Fire burn	Hu	L	F	De	Crush and tie	F	Spr	GC039
*Heteromorpha arborescens* (Spreng.) Cham. &Schldl.	Apiaceae	Yegib mirkuz	S	Snake bite	Hu	R	F	De & O	Chew, absorb and swallow or tie fresh on neck	Fal	Rr	GC015
*Hibiscus macranthus* Hochst. ex A.Rich.	Malvaceae	Nacha	S	Wound	Hu	L	F	De	Chew and cream with cotton	F	Spr	GC064
*Huernia macrocarpa* (A.Rich) Sprenger	Asclepiadaceae	Yemidir kulkual	H	General medicine	Li	Ag	F	O	Chop and give or chop and give after baking with black barley	Fwl	Rr	GC100
*Hypericum quartinianum* A.Rich	Hypericaceae	Amujia	S	Urinary problem	Hu	R	D	O	Crush, powder then eat with honey	F	Spr	GC046
				Stomachache	Hu	L	F	O	Chew and absorb the liquid			
*Indigofera arrecta* Hochst. Ex A. Rich.	Fabaceae	---------	H	Snake bite	Hu	R	F	O	Chew and absorb the juice	Fal	Rr	GC033
*Indigofera prieureana* Guill &Perr.	Fabaceae	-----------	H	Anthrax & Stomach ach	Hu	R	F	O	Chew and swallow juice or crush and give with water	Fal	Spr	GC125
*Jasminum abyssinicum* Hochest. ex DC.	Oleaceae	Tenbelel	S	Toothache	Hu	R	F	O	Take with teeth	F	Wy	GC012
				Snake bite	Hu	Sh	F	O	Crush and drink with water			
*Jasminum grandiflorum* L.	Oleaceae	Terhareg	Cl	Evil eye	Hu	R	FD	De & O	Sniff, drink and fumigate with concoction	F	Spr	GC085
*Juniperus procera* Hochst ex. Engl.	Cuppressaceae	Tid	T	Urine retention	Hu	Fr	FD	O	Boil with TEJ then drink	F	Spr	GC185
				Scrotum swelling	Hu	Gm	F	De & O	Cream			
*Justicia schimperiana* (Hochst. ex Nees) T.Anders.	Acanthaceae	Smiza	S	Wound	Hu	L	DF	De	Crush and powder then cream	Ah	Wy	GC154
				External parasite	Li	L	F	De	Wash with fresh part			
				Anthrax	Hu	Sh	F	O	Crush, mix with water then drink the juice			
				Diarrhea	B	L	F	O	Smash, mix with water then drink the juice			
				Common cold & Hasma	Hu	L	F	Na	Sniff unprocessed or after rubbing			
				Jaundice	Hu	L	F	De &Na	Boil and fumigate			
				Tape worm	Hu	L& St	F	O	Drink the concoction			
				Evil eye	Hu	R	DF	Na, O & De	Sniff, drink and fumigate concoction			
				Rabies	Li	R	F	O	Pound and give with water			
				Stomachache	Hu	L	F	O	Crush and then drink juice			
*Kalanchoe laciniata* L.	Crassulaceae	Endahula	H	General medicine	Li	R	F	De	Peel, tie with tiny rope then insert through skin on neck region	Fwl	Wy	GC084
				Swelling	Li	Ag	F	De	Heat and immediately touch part while hot			
				Febrile illness	Li	R	F	O	Crush and give with water			
				Tape worm	Hu	Ap	F	O	Boil with *Cicer arietinum* cotyledons and eat cotyledons or crush and mix with butter and drink			
*Lactuca intermis* Forssk.	Asteraceae	Dememerarit	H	Broken bone	B	R	DF	De	Tie on the problematic part	Fal	Wy	GC118
				Amoeba	Hu	R	F	O	Chew and swallow the juice			
				Wound	B	Lx	F	De	Cream after removing the ticks			
*Laggera tomentosa (*Sch.Bip. ex A. Rich.) Oliv. & Hiern	Asteraceae	Keskeso/Shetie	H	Swelling	Hu	L	DF	De	Rub and tie or dry, crush , mix with honey and lemon juice then tie	Fwl	Wy	GC038
*Laggera crispata* (Vahl) Hepper & Wood	Asteraceae	Keskesso/ alshasume	H	Gastric & Stomachache	Hu	L	F	O	Chew and swallow the juice	Fal	Wy	GC075
				Tape worm	Hu	L	F	O	Crush and drink with water			
				Stop blood flow after birth	Hu	R	F	De	Crush, immerse in water then spray on newly delivered mother			
				Fire burn	Hu	L	F	De	Rub, squeeze then cream with cotton			
*Leonotis ocymifolia* (Burm.f.) Iwarsson	Lamiaceae	Ferezeng	S	Snake bite	Hu	R	F	De	Crush and tie	F	Rr	GC105
*Leucas martinicensis* (Jaq) R.Br.	Lamiaceae	--------------	H	Prevent diseases relapse	Hu	Ag	DF	De	Fumigate the fume	F	Rr	GC053
*Linum usitatissimum* L.	Linaceae	Telba	H	Wound	Hu	R	D	De	Crush, mix with honey then cream	Fal	Spr	GC184
*Maesa laceolata* Forssk.	Myrsinaceae	Kilabo	S	Womb	Hu	Fr	D	Va	Roast, grind, mix with butter then cream	F	Spr	GC068
*Malva verticillata* L.	Malvaceae	Elit	H	Scabies	Hu	Ag	DF	De	Crush, powder and tie	Ah	Rr	GC103
*Melia azedarach* L.	Meliaceae	Nim	T	Dandruff	Hu	L	F	De	Crush and cream	Hg	Spr	GC160
				Anti-insecticide	Hu	L	DF	De	Crush and powder, then spray with water			
*Millettia ferruginea* (Hochst.) Bak.	Fabaceae	Birbira	T	Leeches	Li	L	F	O	Crush and give with water	F	Rr	GC067
				Rabies	Li	St	DF	De	Heat stick then touch their body with hot part			
*Mimusops kummel* A.DC.*	Sapotaceae	Eshe	T	Hasma	Hu	Fr	F	O	Eat raw fruit	Ris	Rr	GC101
*Momordica foetida* Schumach.	Cucurbitaceae	Yekurahareg/Kuramechat	H	Diarrhea & gonorrhea	Hu	L	F	O	Pound, squeeze then drink	F	Spr	GC165
				Tonsillitis	Hu	L	F	O	Pound, squeeze then drink			
				Sun stroke	Li	L	F	O	Crush and give with water			
				Evil sprit	Hu	L& R	F	De	Boil and fumigate			
*Myrica salicifolia* Hochst. ex A. Rich.	Myricaceae	Shinet	T	Common cold & bleeding	Hu	B	FD	Na	Crush, powder then sniff	Ris	Rr	GC106
				Eye problem	Li	B	FD	Op	Crush, powder then insert			
*Nicandra physaloides* (L.) Gaertn.	Solanaceae	Kassa	H	Fire burn	Hu	L	F	De	Crush, mix with butter then cream	Fal	Spr	GC065
*Nicotiana tabacum* L.	Solanaceae	Tinbaho	S	Wound	Hu	L	D	De	Crush and powder then cream	Hg	Rr	GC080
*Nuxia congesta* R.Br. ex Fresen*.*	Loganiaceae	Atquar	S	Tonsillitis	Hu	Sh	F	De & O	Rub, squeeze then drink and put on head	F	Spr	GC088
*Ocimum urticifolium* Koth	Lamiaceae	Dama kesie	S	Febrile illness	Hu	L	F	O	Boil with tea and drink	Hg	Spr	GC129
				Common cold	Hu	L	F	O	Boil with tea and drink			
*Olea europaea* L. subsp. *cuspidata* (Wall. ex G. Don) Cif.	Oleaceae	Woira	T	Tonsillitis	Hu	L	F	O	Chew and absorb the juice	Ft	Wy	GC079
				Evil eye	Hu	St	F	De	Beating with fresh stick			
				Eye diseases	Hu	L	F	Op	Pound, squeeze then drop with cotton			
				Deafness	Hu	L	F	Et	Drop concoction with food oil			
*Ormocarpum pubescens* (Hochst.) Cuf.ex.Gillett	Fabaceae	Murna	S	Wound	Hu	L	DF	De	Crush, powder then tie	F	Rr	GC014
*Orobanche ramosa* L.	Orobanchaceae	------	H	Sunstroke	Li	Ap	D	De	Fumigate	Fwl	Rr	GC181
*Otostegia integrifolia* Benth.	Lamiaceae	Tunjut	S	Epidemic & common cold	Hu	Ag	D	De	Fumigate the house	F	Spr	GC141
				Coccolida	Li	Ag	D	De	Fumigate			
				Stomachache	Hu	Sh	F	O	Rub, squeeze then drink liquid			
*Pentas lanceolata* (Forssk.) Defl.	Rubiaceae	Ras faris	S	Tite problem	Li	L	F	De	Crush, powder then cream	F	Rr	GC066
*Periploca linearifolia* Quant. Dill. & Rich.	Asclepiadaceae	Moider	Cl	Hemorrhoid	Hu	St	F	De	Heat with fire then immediately apply	F	Spr	GC150
				Hemorrhoid	Hu	R	F	De	Crush and tie			
*Persea americana* Mill.	Lauraceae	Avocado	S	Kidney infection	Hu	L	F	O	Boil and drink juice	Hg	Rr	GC183
*Phyllanthus rotundifolius* Willd.	Euphorbiaceae	-----------	H	Ring worm	Hu	Lx	F	De	Cream	Fal	Rr	GC019
*Phytolacca dodecandra* L’Herit.	Phytolaccaceae	Endod	S	Leeches	Li	L	F	Na	Crush and insert with water	Bo	Spr	GC024
				Jaundice	Hu	L	F	O	Crush and drink with water			
				External parasite	Li	L	F	De	Wash with unprocessed leaf			
				Rabies	Li	R	F	O	Crush and give with milk			
				Elephantiasis	Hu	L	F	De	Crush, decant, and insert juice			
				Malaria	Hu	R	F	O	Crush, squeeze then drink			
				Anthrax	Hu	Sh	F	O	Crush, mix with water then drink			
				Coccinia	Li	R	F	O	Crush, immerse in water then give			
*Plantago lanceolata* L.	Plantaginaceae	Wonberet/ Gorteb	H	Wound & bleeding	Hu	L	DF	De	Crush, powder then cream	Fal	Wy	GC117
*Plectranthus tenuiflorus* (vatke) Agnew	Lamiaceae	Mutansa	S	Weaken babies & evil sprit	Hu	Ap	DF	O	Crush, powder then give with water	Hg	Rr	GC148
*Plumbago zeylanica* L.	plumbaginaceae	Amera	H	Wound	Hu	R	DF	De	Cream concoction	Fwl	Rr	GC128
				Stomachache &Scorpion poison	Hu	L& R	F	O	Crush and drink with water			
*Premna schimperi* Engl.	Lamiaceae	Chocho	S	Eye problem	Li	L	F	Op	Chew and spit	F	Spr	GC126
				Wound	Hu	B & L	D	De	Crush, powder then cream with butter or honey			
				Toothache	Hu	R	F	O	Chew and take with teeth			
*Prunus persica* (L.) Batsch	Rosaceae	Kok	S	Diarrhea	Li	L	F	O	Crush, immerse in water then give	Hg	Rr	GC049
				Tape worm	Hu	L& St	F	O	Drink the concoction			
*Punica granatum* L.	Punicaceae	Roman	S	Cancer & skin diseases	Hu	Fr	F	O	Crush and eat	Hg	Pa	GC022
*Rhamnus prinoides* L’Herit	Rhamnaceae	Gesho	S	Tonsillitis	Hu	Sh	F	O	Crush and drink with water	Hg	Spr	GC094
				Herpes	Hu	L	F	De	Grind and cream			
*Ricinus communis* L.	Euphorbiaceae	Chakima/ Gulo	**S**	Calf diarrhea	Li	Fr	F	O	Pound cream the teat of cow then allow to suck	Hg	Rr	GC170
*Rosa abyssinica* Lindley*	Rosaceae	Kega	S	Tension/dizziness	Hu	Fr	F	O	Eat the raw fruit	F	Spr	GC037
*Rubia cordifolia* L.	Rubiaceae	Mencherer	Cl	Cough	Hu	R& L	F	O	Drink the concoction with tea or coffee	F	Rr	GC110
*Rumex abyssinicus* Jacq.*	Polygonaceae	Mekmoko	H	Hypertension	Hu	R	DF	O	Pound, powder then drink with milk	Fal	Spr	GC076
*Rumex nepalensis* Spreng.	Polygonaceae	Tult	H	Tonsillitis & diarrhea	Hu	R	DF	De	Crush, mix with water then drink juice or tie on neck without processing	Fwl	Spr	GC029
				Stomachache	Hu	R	DF	O	Chew and swallow the juice			
				Anthrax	Li	R	F	O	Crush and give with water			
*Rumex nervosus* Vahl*	Polygonaceae	Enbuacho	S	Wart	Hu	L	F	De	Rub, squeeze then cream	Fal	Wy	GC177
				Bleeding wound	Hu	L	F	De	Pound then tie			
*Ruta chalepensis* L.	Rutaceae	Tenadam	H	Evil eye	Hu	L	DF	De & O	Sniff, drink and fumigate with concoction	Hg	Rr	GC186
				Febrile illness	Hu	L	F	O	Crush then fumigate whole body or drink the concoction			
*Sansevieria erythraeae* Mattei	Dracaenaceae	Chiret	S	Ear wound	Hu	St	F	Et	Heat, pound, squeeze then insert while cool	Hg	Rr	GC111
*Schefflera abyssinica* (Hochst. ex A. Rich.) Harms.	Araliaceae	Getem	T	Snake poison	Hu	B	F	O	Crush and drink the infusion	F	Rr	GC171
*Schinus molle* L.	Anacardiaceae	Kundoberbere	T	Cough	Hu	Fr	DF	O	Pound, cook in DORRO WOTE then eat with TEF ENGERA	Hg	Spr	GC155
				Wound	Hu	L	F	De	Pound and tie			
*Senna didymobotrya* (Fresen.) Irwin &Bameby	Fabaceae	Serka Abeba	S	Bloating	Li	L	F	O	Crush and give with water	Fwl	Wy	GC122
*Sida ovata* Forssk.	Malvaceae	Yahya-nacha	H	Fire burn	Hu	R	F	De	Pound and cream the liquid with cotton	Fal	Spr	GC032
*Sida rhombifolia* L.	Malvaceae	Gorgegit	S	Impotency	Hu	R	F	O	Drink concoction with honey	Bo	Spr	GC120
				Wound	Hu	L	F	De	Crush and tie			
*Sida tenuicarpa* Vollesen	Malvaceae	Chifrig	S	Wound	Hu	L	F	De	Crush and tie	Fwl	Spr	GC153
				Evil spirit & evil eye	Hu	R	DF	De & O	Used as tooth brush or tie on neck			
*Solanecio gigas* Vatke	Asteraceae	Yashikoko gomen	S	Bloating	Li	L	F	O	Pound and give with water	Hg	Pa	GC061
				Evil eye	Hu	R	DF	Na, O & De	Sniff, drink and fumigate with concoction			
*Solanum anguivi* Lam.	Solanaceae	Zerch enboy	S	Wound	Hu	L	DF	De	Crush, pound and tie	F	Spr	GC174
				Wart	Hu	Fr	F	De	Cream with juice			
				Beating with stick	Li	R	F	O	Crush and give the infusion			
*Solanum incanum* L.	Solanaceae	Yekolla enboy	S	Stomachache	Hu	R	F	O	Crush, chew then absorb juice	Fwl	Spr	GC059
				Ring worm	Hu	Fr	F	De	Heat fruit then cream with juice			
				Wart	Hu	Fr	F	De	Cream with juice			
				Arthritis/rheumatism	Hu	L	F	De	Pound and tie			
				Leeches	Li	Fr	F	Na	Insert juice			
				Diabetic	Hu	R	F	O	Chew and swallow juice			
				Febrile illness	Li	R	F	O	Pound and give with water			
				Wound	Hu	Fr	F	De	Cream with juice			
				Scorpion poison	Hu	Fr	F	O	Drink juice with water			
*Solanum marginatum* L.f.	Solanaceae	Yedega enboy	S	Cough	Li	Fr	F	Na	Give juice with goat milk	F	Rr	GC095
*Solanum nigrum* L.*	Solanaceae	Awut	H	Spider poison	Hu	L	F	De	Crush, squeeze then cream	Fwl	Rr	GC140
				Hemorrhoid	Hu	Ap	DF	De	Pound and tie			
				Diarrhea	Hu	L	F	O	Crush, chew then swallow juice			
*Steganotaenia araliacea* Hochst. ex A.Rich.	Apiaceae	Endoka/Yefiyel chew	T	Hemorrhoid	Hu	St	DF	De	Peel, heat then apply in the hot condition	F	Spr	GC083
*Stephania abyssinica* (Dillon & A. Rich.) Walp.	Menispermaceae	Chewchawit	H	Anthrax	B	R	F	O	Crush and give with water	Fal	Spr	GC121
				Anthrax & Stomachache	Hu	R	F	O	Chew and swallow the juice			
				Rabies	B	R	F	O	Crushed and given with milk and water			
				Tonsillitis	Hu	Sh	F	O	Crush and drink with water or cream on neck region			
*Stereospermum kunthianum* Cham.	Bignonaceae	Zana	T	Eye problem	Li	B	DF	O	Cream the concoction with butter and apply to cattle	F	Spr	GC017
				Scorpion & Snake poison	Hu	B	F	De	Pound and tie or chew and swallow the juice			
*Striga hermonthica* (Del.) Benth.	Scrophularaceae	Gelmit	H	Bloating	Li	Ap	DF	O	Crush, powder and give with water	Fal	Spr	GC144
*Syzygium guineense* (Willd.) DC.*	Myrtaceae	Dokima	T	Diarrhea	Hu	B	F	O	Crush and drink with water	Ris	Spr	GC045
*Thalictrum rhynchocarpum* Dill. & A. Rich.	Ranunculaceae	Sire-bizu	H	Scrotum swelling	Hu	R	F	De	Crush and drink with TELLA	F	Rr	GC078
				Impotency	Hu	R	F	O	Drink concoction with honey			
*Tragia brevipes* Pax.	Euphorbiaceae	Abelbalit	H	Swelling	Hu	R	F	De	Pound and tie	F	Rr	GC013
				Impotency	Hu	R	F	O	Drink concoction with honey			
*Urera hypselodendron* (Hochst.) ex A. Rich.	Urticaceae	Lankusso	Cl	Anthrax	Li	Sh	F	O	Crush and give with water	F	Spr	GC 060
*Urtica simensis* Steudel	Urticaceae	Sama	H	Gastric	Hu	L	F	O	Roast, grind and drink juice	F	Rr	GC 179
				Wound	Hu	L	F	De	Grind and cream with butter			
*Verbasicum sinaiticum* Benth.	Scrophularaceae	Kutitina	S	Stomachache	Hu	R	F	O	Pound and drink with honey or water or butter	F	Spr	GC074
				Diarrhea	Hu	R	F	O	Crush and drink with water			
				Evil sprit	Hu	L	F	De	Boil and fumigate with the fume			
				Evil eye	Hu	R	DF	Na, O & De	Sniff, drink and fumigate concoction			
*Verbena officinalis* L.	Verbenaceae	Atuch	H	Bleeding	Hu	R	F	De	Crush and tie	Fal	Wy	GC069
				Evil spirit & intestinal poison	Hu	Ag	DF	O	Crush and drink with water			
				Evil eye	Hu	R	DF	Na & O	Sniff, drink and fumigate concoction			
				Tonsillitis	Hu	Ap	F	O	Crush and drink with water			
				Impotency	Hu	R	D	O	Drink concoction with honey			
				Deafness	Hu	L	F	Et	Pound and ingest juice with water			
				Stomachache & Anthrax	Hu	R	F	O	Chew and swallow the juice			
*Vernonia adoensis* Sch.Bip ex Walp.	Asteraceae	Eras abera/ Este musaye	S	Likfit (skin rash)	Hu	R	F	De	Crush. powder then cream with butter	Hg	Spr	GC147
				Amoeba, Gardiasis, Gastric & Snake poison	Hu	R	F	O	Crush, powder then drink with water or Chew and swallow juice			
*Vernonia amygdalina* Del.	Asteraceae	Girawa	S	Bloating	Li	L	F	O	Crush and give with water	Hg	Rr	GC055
				Dandruff	Hu	L	F	De	Pound and cream			
				Impotency	Hu	R	F	O	Drink the concoction with tella			
*Vernonia myriantha* Hook.f.	Asteraceae	Kotkoto	S	Impotency	Hu	R	DF	O	Drink the concoction with tella	Fwl	Wy	GC057
*Vicia faba* L.	Fabaceae	Bakela	H	Anemia	Hu	Sd	D	O	Roast and drink infusion	Hg	Spr	GC109
*Withania somnifera (*L.) Dunal in DC.	Solanaceae	Giziewa	S	Evil eye & evil sprit	Hu	L & R	DF	O	Crush and drink with water or fumigate with the fume	Hg	Rr	GC048
				Tape worm & Babies disease	Hu	L	DF	De	Fumigate in a closed fashion			
				Cough	Hu	L	F	O	Crush and boil with milk then drink			
				Impotency	Hu	R	F	O	Drink concoction with honey			
*Xanthium strumarium* L.	Asteraceae	Gid zemede	H	Dandruff	Hu	L	F	De	Rub, squeeze then cream	Fwl	Spr	GC112
*Ximenia americana* L.*	Olacaceae	Enkoy	S	Wound	Hu	B	DF	De	Crush, grind and cream	F	Rr	GC054
*Zea mays* L.	Poaceae	Mashilla	H	Dandruff	Hu	Sw	F	De	Burn and cream ashes with butter	Hg	Wy	GC030
*Zehneria scabra* (Linn. f.) Sond.	Cucurbitaceae	Hareg resa	Cl	Swelling	Hu	L	F	De	Crush and tie	Ah	Rr	GC149
				Wound	Li	Ag	F	De	Rub and cream			
				Febrile illness	Hu	Ag	F	De	Boil and take the fume in enclosed fashion			
				Diarrhea	Hu	L	F	O	Crush, chew then swallow juice			
*Ziziphus spina-christi* (L.) Desf. *	Rhamnaceae	Gava	T	Dandruff	Hu	L	F	De	Pound and cream	Hg	Pa	GC163

**Key**: **Parts Used:** B (stem bark), Rb (root bark), R (root), L (leaf), Ap (all part), St (stem), Bu (bulb), Lx (latex), Fl (flower), Sd (Seed), Sh (shoot), Fr (fruit), Sp (Sap), Sw (Straw), Gm ( gum), Ag (above ground); **Growth forms (Gf)**:-S (shrub), T (tree), Cl (climber), H (herb), P (parasite); **Ailment type (At):** Hu (human) LI (livestock); **CP (condition of preparation):** F (fresh), D (dry), DF/FD (dry and fresh); **Route of administration (Ra):** De (Dermal), O (Oral), Na (nasal), Op (Optical), Va (Vaginal), Et (Ear tube); **Habitat (Ha): Wild** (F (Fores), Fal (Farm land), Fwl (Fallow land), Rs (Road side), Ris (river side), Ah (around home)), Aw (All wild type of habitats i.e Forest, Farm land, Fallow land, Road side, river side and around home), Bo (all wild type habitats and homegarden), Hg (Homegarden), **Distribution(Dn):** Spr (Sparse), Wy (Widely), Rr (Rare), Pa (Particular area); Co. No.(Collection number) *Wild food plant species.

**Table 5:** **The six most acclaimed medicinal plants based on informant citation**

Scientific name	Ailments claimed to treat	No. of citations	Percentage	Rank
*Zehneria scabra*	Diarrhea, wound, febrile illness and swelling	60	57.14	1^st^
*Stephania abyssinica*	Human and livestock anthrax, tonsillitis, rabies and stomachache	55	52.40	2^nd^
*Otostegia integrifolia*	Stomachache, hen’s coccolida, epidemic diseases and common cold	40	38.10	3^rd^
*Verbascum sinaiticum*	Stomachache, diarrhea, evil eye & evil sprit	32	30.47	4^th^
*Capparis tomentosa*	Evil eye, and epidemic diseases	27	25.71	5^th^
*Achyranthes aspera*	Tape worm, wounds, excessive menstrual flow, tonsillitis, bleeding, bone fracture, and eye problems	25	23.80	6^th^

**Figure 2:**
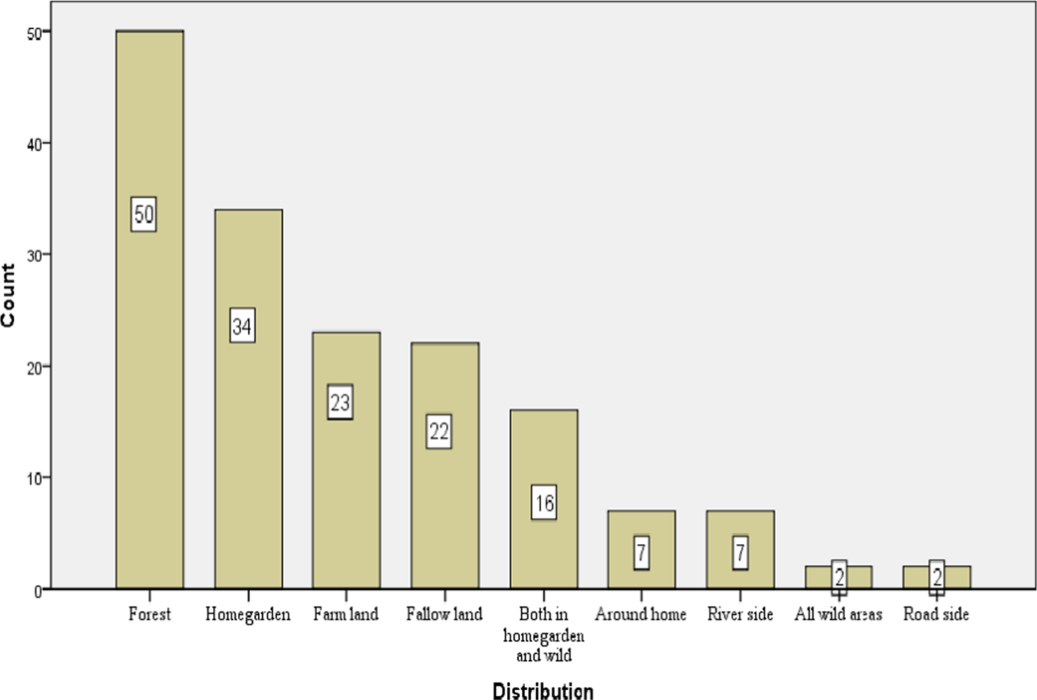
**Distribution of medicinal plant species in different habitats.**

Among the reported medicinal plants of the area, some were also reported as wild edible plants (Table 4). Informants, during data collection, said that some of the species for example, the edible parts (fruits) of *Rosa abyssinica* are used to alleviate weakness or tension when eaten by children in the field. This is done without knowing the medicinal effects of the plants and those who eat it feel happy and accomplish their tasks effectively. Herbs accounted for 67 (41.1%) species followed by shrubs (62, 38.0%), trees (24, 14.7%) and climbers (10, 6.1%). The medicinal plants occur in the wild, homegardens and in both premises. The forests, farmlands, margins, living plants on fences, roadsides, around homes, fallow lands and riversides are the habitats where the medicinal plants are found (Figure 2).

### Health disorders treated and ICF

The analyses on application of plants showed that 115 (70.6%) species in 103 genera and 54 families were listed as medicines for human ailments, 34 (20.9%) species in 32 genera and 22 families for both human and livestock ailments and 14 (8.6%) species in 14 genera and 11 families were reported as medicine for livestock ailments only. These medicinal plants were claimed to be of use in the treatment of about 60 types of human ailments only, 10 types of both human and livestock health disorders and nine types of livestock ailments only. For the most common ailment (wound), 42 medicinal plant species were reported (Table 7). The ailments were classified into 13 categories and ICF values were computed and livestock ailments had the highest ICF value of 0.84 and other disease categories had lower values (Table 8).

**Table 6:** **Single medicinal plant species prescribed for treatment of higher number of ailments**

Plant species name	No. of ailment treated	Plant species name	No. of ailment treated
*Justicia schimperiana*	11	*Achyranthes aspera*, *Cucumis ficifolius* and *Euphorbia abyssinica*	7 each
*Croton macrostachyus*, *Verbena officinalis and Solanum incanum*	9 each	*Ferula communis*, *Cynoglossum*, *coeruleum*, *Asparagus africanus*, *Calpurnia aurea*	6 each
*Phytolacca dodecandra*	8

**Table 7:** **The most common disease with their respective number of medicinal plant species**

S.no	Ailments	No of species for each ailment	S.no	Ailments	No of species for each ailment
1	Wound	42	6	Impotence	11
2	Stomachache	25	7	Tonsillitis, rabies, hemorrhoid, fibril illness, and snake bite	10
3	Intestinal parasites	23	8	Dandruff	8
4	Anthrax	16	9	Livestock bloating and malaria Common cold and cough	6
5	Diarrhea	13			

### Importance of the medicinal plants

Some medicinal plants were rated as important and used frequently by many, appearing in many formulations. Preferences for six common medicinal plant species said to be used for the treatment of the common ailment (wound) showed *Cordia africana* in the first rank order followed by *Sida rhombifolia* (Table 9). The pair-wise comparison of medicinal plants used for the treatment of stomachache showed that *Stephania abyssinica* was the most reported and ranked first, while *Otostegia integrifolia* was the least ranked plant species (Table 10). Matrix ranking of six popular multipurpose medicinal plants showed that *Cordia africana* was the most useful multipurpose medicinal plant that was ranked 1^st^ while *Croton macrostachyus* was the least ranked one (Table 11).

**Table 8:** **ICF value for each disease category**

Disease categories	Nu	Nur	Fic
Livestock diseases (external parasites, beating with stick and sun stroke)	16	94	0.84
Febrile illness, headache, anemia, brain tension and malaria	19	80	0.78
Rabies	11	46	0.76
Gastrointestinal disorders	52	205	0.75
Dermal diseases (wound and skin diseases)	72	221	0.68
Bone fracture and Arthritis	7	18	0.65
Reproductive and sexual organs	22	61	0.65
Bleeding and hypertension	7	14	0.54
Respiratory diseases (asthmatic reactions, cough, common cold, leech and tonsillitis)	24	48	0.51
Sense organs like eye and ear problems	21	42	0.51
Spider, snake, and scorpion poisons and bites	18	32	0.45
General disease (tension, epidemic, baby diseases and undefined diseases)	28	47	0.41
Organ diseases (diabetes, heart problem, jaundice, kidney infection, pneumonia, urinary problem)	12	16	0.26
Anthrax, cancer and hemorrhoid	24	25	0.04

**Table 9:** **Simple preference ranking of six medicinal plants used against wound in the study area**

Medicinal plant species	Respondents (R1- R7)								
	R _1_	R _2_	R _3_	R _4_	R _5_	R _6_	R _7_	Total	Rank
*Brucea antidysenterica*	5	5	1	4	6	5	3	29	3^rd^
*Cordia africana*	6	6	5	5	5	6	6	39	1^st^
*Dodonaea angustifolia*	3	2	6	1	4	3	1	20	4^th^
*Ficus carica*	2	1	3	3	1	2	2	14	6^th^
*Plantago lanceolata*	1	3	2	2	2	1	4	15	5^th^
*Sida rhombifolia*	4	4	4	6	3	4	5	30	2^nd^

**Table 10:** **Paired comparison on five medicinal plants used to treat stomachache in the study area**

Medicinal plants used	Respondents (R1- R7)								
	R _1_	R _2_	R _3_	R _4_	R _5_	R _6_	R _7_	Total	Rank
*Cucumis ficifolius*	1	2	1	2	2	2	1	11	4^th^
*Indigofera prieureana*	2	2	3	2	3	3	2	17	2^nd^
*Otostegia integrifolia*	0	1	0	2	1	1	3	8	5^th^
*Stephania abyssinica*	4	4	4	4	1	3	2	22	1^st^
*Verbascum sinaiticum*	3	1	2	0	3	1	2	12	3^rd^

### Plant parts used and modes of remedy preparations

Out of the total plant parts used for remedy preparation, leaves were the highest (109, 31.2%), followed by roots (108, 30.9%) and lower values for other parts (Table 12). Information about the preparation of each plant has been included in Table 4. The results also showed that the majorities of remedies (89%) were prepared from single plant species and few (11%) were prepared from combinations of more than two medicinal plant species. Simple modes of preparation of medicine including crushing (90.5% informants), chewing, pounding, chopping and juice extraction were used (Table 13).

**Table 11:** **Matrix ranks of six multipurpose medicinal plants in the study area**

Plant species name	Medicine	Cash income	Fuelwood	Food	Forage/ fodder	Construction/ building	Shade	Total	Rank
*Carissa spinarum*	5	4	5	4	4	2	1	25	2^nd^
*Cordia africana*	4	5	3	5	5	2	3	27	1^st^
*Croton macrostachyus*	4	1	2	0	1	2	5	15	6^th^
*Ficus sur*	2	3	2	5	4	2	5	23	4^th^
*Mimusops kummel*	2	4	1	5	2	2	5	21	5^th^
*Olea europaea* ssp. *cuspidata*	3	5	5	0	4	5	2	24	3^rd^

**Table 12:** **Frequency of plant parts used for the preparation of remedies**

Plant parts used	No. of preparations	Percentage	No. of species	Plant parts used	No. of preparations each	Percentage	No. of species
Leaf	109	31.2	56	Stem	6	1.7	4
Root	108	30.9	45	Bulb	5	1.4	1
Fruit	25	7.2	13	Flower	4	1.1	3
Bark	15	4.3	11	Sap	1	0.3	1
Shoot	15	4.3	5	Gum	1	0.3	1
Latex	13	3.7	6	All parts	10	2.9	7
Seed	7	2.0	5	Two and three parts	15	4.3	13

### Condition of preparation and storage of plant medicines

The results of the analyses showed that 70.94% of the plant medicines were prepared from fresh plant parts, 9.69% from dried and 19.37% from both fresh and dried parts. Healers explained that some of the stored remedies were kept for about one year, from September to September of the next year and discarded on the Ethiopian New Year and replaced with new preparations. When a particular medicinal plant could not be accessed easily, the previously stored remedy would be buried in the ground for one day (from the eve of the end of the first day of the New Year), after which time it is declared safe to be used. It was explained that remedies were stored secretly in a very secure place (mostly outside the living house at the top of the wall to keep them far from children) and no one is allowed to touch them without permission.

### Route of remedy administration and dosage determination

It was found that the local people employ about 10 ways of medicine administration routes with varying frequencies. Of the total, 157 (44.9%) prescriptions were mainly those said to be applied through oral route (Table 14). The dosage varied between age and patient’s capacity as judged by healers. Traditional ways of dosage determination included measurements, namely, atq (referring to the size of the finger stripe/line, mostly of the small finger), tfir (referring to the size of a fingernail), finjal (referring to the volume of the coffee cup), birchiko (referring to the volume of a glass, mostly of tea glass). And tassa (referring to the volume of a tin can), mankia (referring to the size of a teaspoon) and faga (referring to a container made from a small fruit of the bottle gourd (*Lagenaria siceraria*) as well as number (leaves, fruits, seeds), size and droplets of plant parts. Smaller sizes (atq and tfir) were used to determine dosages of the most toxic plants including *Euphorbia abyssinica*, *Stephania abyssinica and Calpurnia aurea*, and the two measurements plus finjal, birchiko and mankia are meant for oral administration of medicine for the treatment of internal human ailments. finjal, birchiko, tassa and faga were used for less toxic plants that were diluted with liquid additives including tea, milk, coffee and water. Remedies were mixed mostly with water, honey, tea, milk, coffee, and dosages prescribed as half, one, two, and so on of materials used per day based on the nature of plants and patient’s age and general condition (body, health). tassa and faga were prescribed for use to treat livestock ailments while faga for preparation and dosage determination for external application of remedies in the cases of both humans and livestock treatment. The concepts of dosage and measurement do exist in the traditional herbal medical system of the community as it emerges from the practices albeit the low precision. Even though the experienced medicinal plant practitioners showed serious concerns in determining the dosages very carefully; the measuring devices they used do not allow delivery of precise amounts. The members of the association of healers and some other local community members reported the effectiveness of traditional medicine, but they expressed discomfort when it comes to the amount given particularly in the case of internal human medicines. They actually recommended that technical assistance and psychological support through training must be given to minimize the fear and effect of incompatible dosage of remedies on patients. The measurements used to determine the dosages are not standardized except categorization by age, physical appearance and health conditions. The absence of adverse effects of traditional herbal medicines after administration was most frequently mentioned by the traditional healers. Coffee and milk were mentioned for use as antidotes when formulations were made from *Euphorbia abyssinica*, for malaria, and *Calpurnia aurea* for diarrhea and anesthesia. Likewise, local beer (tella) is used as antidote when *Asparagus africanus* is used to treat impotence. The traditional healers indicated that they use the antidotes for dilution in cases of adverse effects.

**Table 13:** **Mode of preparation of medicinal plants**

Types of preparation	Frequency of preparation	Percentages
Crushing	118	35.01
Grinding, concoction and creaming	50	14.80
Boiling, heating, burning and fumigation	49	14.50
Chewing, spitting and absorbing fluid/juice	32	9.50
Rubbing and squeezing	24	7.10
Using unprocessed plant part	23	6.80
Pounding and making infusion	23	6.80
Chopping and breaking	18	5.30

### Marketed medicinal plants in the study area

Survey of two towns in the proximity of the study sites (Addis Zemen and Yifag) did not show any medicinal plant mentioned during the interviews presented on the market. The respondents explained that most healers prepared and sold traditional medicinal plants in the home rather than in the open market. Healers usually had big signposts in front of their homes listing the health problems they treat. Some medicinal plants were marketed mainly for other use values (spices and food) but once bought they could be used as medicine at home as part of the common family home treatment. These include *Allium sativum*, *Ruta chalepensis*, *Brassica carinata* and Cicer *arietinum* usually traded for use as edible spices. On the other hand, *Carica papaya*, *Citrus aurantifolia*, *Citrus aurantium*, *Coffea arabica*, *Cucurbita pepo*, *Linum usitatissimum*, *Mimusops kummel*, *Persea americana*, *Prunus persica*, *Punica granatum*, *Zea mays*, *Eragrostis tef*, *Capsicum annuum* and *Vicia faba* were bought from the market for use as food items.

### Taboos connected with handling and use of medicinal plants

Some of the taboos reported by experienced medicinal plant experts concern times of collection, ways of collection, preparation materials, administration and storage. Most of the medicinal plants were said to be collected on Wednesdays and Fridays in the early morning hours without contact and without talking to any other person and this is related to healers’ beliefs that doing it otherwise would reduce the efficacy of the herbal medicine. In the preparation of a single remedy, plant parts are mostly taken from individuals of the same species growing in three or seven different places. One healer said that this increases its remedial effectiveness. This could be a way of balancing the amount of phytochemical and pharmacological constituents based on habitat variation. Collection materials are kara (kind of knife), ankasie/tore (metallic spear), weyra ejeta mekoferia (digger with handle made of *Olea europaea* ssp. *cuspidata* wood) and most of the time stationary stones are the preferred preparation places. It was mentioned that sexual intercourse is forbidden for healers and patients alike during any medicinal plant collection, preparation and application.

### Variation of indigenous plant knowledge in the study area

**Figure 3:**
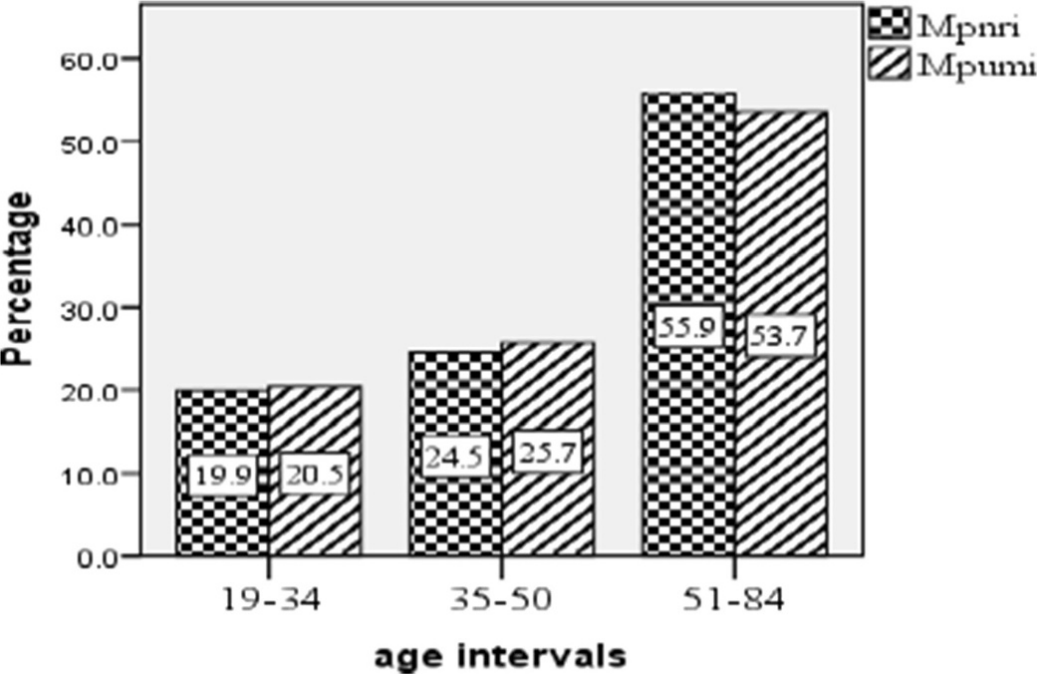
**Variation of medicinal plants knowledge among the age groups of informants** **(Mpnri** **=** **Medicinal plant names reported by individuals,** **Mpumi** **=** **Medicinal plant uses mentioned by individuals).**

Significant correlation (Spearman correlation test, r = -0.450, α = 0.05, p = 0.046) was observed between male and female informants on the number of medicinal plant species they knew. The test, however, did not indicate significant correlation between healers and general informants (Spearman correlation test, r = -0.002, α = 0.05, p = 0.991) regarding the number of medicinal plant species they reported. The comparison of knowledge and experience of age groups (35–50 and 51–84) showed significant differences (P < 0.05) while there was no significant difference between age groups 19–34 and 35–50 considering plant names and the respective medicinal uses (t = 0.05, two tailed and df = 52). Progressively increasing results were obtained with increasing age of informants (Figure 3).

Local community members in Washa Indiras, Kidanemhret and Kualla Yihuans gave 162, 95 and 91 medicinal plant names with 128, 95 and 86 medicinal uses respectively. Informants from Washa Indiras village reported the highest plant names (162) and uses (128), while those in Yifag Akababi and Asiba turned in the least numbers (58, 56) and uses (52, 50) respectively. However, not all communities living nearby the forests gave higher reports compared to distant villages. For example, Tibabosgie is the nearest village to the forest, but the report from informants showed relatively lower names (48) and uses (50) than the other villages found relatively far from the forest, namely Abuarra (92 names and 80 uses), Lomye (73 names and 80 uses) and Agamoch (69 names and 66 uses). On the other hand, Mantogera village is located nearest to the forest, but the results showed 61 names and 61 uses, which is less than other nearby villages in the same (woina dega) agroclimatic zone. Furthermore, in Aguat Mafsesha located at higher altitude of all villages found in dega agroclimatic zone, showed that informants could only recall a few species and uses (40 names and 43 uses). Generally, however, informants in villages near the forest knew more plants (38.5%) and uses (38.9%) than those located in towns (30.3%, 30.0%) and far away from forests (30.8%, 31.5%).

### Indigenous medicinal plant knowledge development and sharing

Traditional knowledge of medicinal plants in most cases is passed along the family line from parents and other intimates, especially gifted family members (which they described as eju yemisemrlet, meaning one whose hands are skillful and effectual). Some of the traditional knowledge is generated through the community by listening and practicing while some copied secretly and systematically by following and observing the knowledgeable individuals at times of medicinal plant collection and preparation. Others develop and transfer their medicinal plant knowledge to generations by following up healers after seeking treatment of their family members. In very few cases, individuals developed their medicinal plant knowledge upon careful observation of domestic carnivores, especially the cat, which immediately consumes medicinal plant parts upon preying on poisonous snakes, scorpions and spiders. One healer reported his discovery in this way of *Vernonia adoensis* for the treatment of snake poison. Medicinal plant experts have developed some traditional medicinal plant knowledge from observations of animal feeding to know the plants that are never consumed, which hints at plants not for internal use to ensure safety of the vital organs but rather used for the treatment of dermal ailments such as wounds because of their possible toxic nature. Furthermore, experienced medicinal plant experts create new medicinal knowledge by relating the plant odour with previously known medicinal plants. Some healers were seen recording ethnomedicinal knowledge in small notebooks during fieldwork, which may testify their curiosity and keenness to develop and transfer indigenous knowledge to the next generation.

### Threats to and conservation of medicinal plants and associated indigenous knowledge

**Figure 4:**
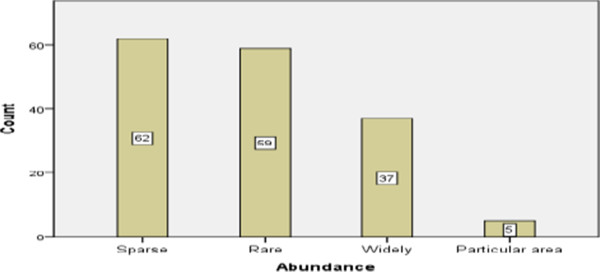
**Current condition of medicinal plant species based on informant preferences.**

The study found that medicinal plants are faced with threats in their habitats. Informants claimed that long before the past ten to twenty years Tara-gedam and the surrounding areas were full of natural vegetation around the farmlands, riversides and grazing lands in addition to the wealth of plant species in number and diversity in the forests. They further asserted that in those days almost all the medicinal plants were easily accessible within short distances of the living place. Today, it is not an easy task to get medicinal plants out of Tara-gedam and Amba forests due to habitat modification. Most informants perceived that agricultural expansion was the main threat to medicinal plants, firewood collection the next and others follow (Table 15). Similarly, preference ranking of five most threatened medicinal plant species indicated that *Withania somnifera and Huernia macrocarpa* are the two most threatened medicinal plants (Table 16). Through further discussion and interview with informants, 63 plant species that were said to have become sparse in distribution were recorded along with five species restricted in occurrence and in most cases found in the homegardens in recent years (Figure 4).

**Table 14:** **Mode of administration of the plant remedies**

Mode of administration	Number of medicinal plant parts used in each case	Percent of total
Oral	157	44.9
Dermal	132	37.7
Dermal, nasal and oral	14	4.0
Dermal and oral	15	4.3
Optical	10	2.9
Nasal	9	2.6
Ear	8	2.3
Vaginal	2	0.6
Dermal and nasal; nasal and ear; nasal and oral	1	0.9

**Table 15:** **Priority ranking results of seven respondents on six factors perceived as threats to medicinal plants**

Threatening factor	Respondents (R1- R7)								
	R _1_	R _2_	R _3_	R _4_	R _5_	R _6_	R _7_	Total	Rank
Agricultural expansion	6	6	6	5	5	6	4	38	1^st^
Overgrazing	3	4	5	6	6	5	3	32	3^rd^
Drought	2	2	2	1	3	4	4	18	5^th^
Fuelwood collection	6	4	6	2	6	4	5	33	2^nd^
Construction and building material	1	3	5	1	1	2	3	15	6^th^
Urbanization/Modernization	4	6	5	2	3	4	5	29	4^th^

**Table 16:** **Results of preference ranking of five most threatened medicinal plants**

Treating medicinal plant species	Respondents (R1- R7)								
	R _1_	R _2_	R _3_	R _4_	R _5_	R _6_	R _7_	Total	Rank
*Cucumis ficifolius*	4	1	4	1	2	2	3	17	3^rd^
*Ficus carica*	3	2	1	2	3	2	3	16	4^th^
*Huernia macrocarpa*	2	3	3	4	4	4	5	25	2^nd^
*Solanum marginatum*	1	2	2	3	1	3	2	14	5^th^
*Withania somnifera*	5	4	5	5	5	5	4	33	1^st^

Conservation efforts specifically targeted to medicinal plants do not exist in the District. However, some of the medicinal plants are raised in the governmental nurseries for other purposes and conserved in the protected governmental and Orthodox Tewahedo church forests. The well known Tara-gedam and Amba natural forests and other relatively smaller patches of vegetation and plantations found in each kebele are nowadays being protected by the local people living around the forest fringes in collaboration with the government. Some of the medicinal plants occurring in the Orthodox Tewahedo church forests were *Adiantum capillus*-*veneris*, *Clerodendrum myricoides*, *Juniperus procera*, *Millettia ferruginea*, *Schefflera abyssinica*, *Urera hypselodendron* and *Ziziphus spina*-*christi*. The informants elaborated that some of the medicinal plants collected from the homegardens namely, *Persea americana*, *Citrus aurantifolia*, *Citrus aurantium*, *Coffea arabica*, *Cordia africana*, *Ficus sur*, *Schinus molle* and *Punica granatum* were those raised from seedlings taken from the nursery. It was also observed that the local farmers make use of their indigenous knowledge in protecting important plant species on their farmlands, homegardens, or as live fence. Few traditional healers cultivate very rare species in their homegardens. Healers mentioned the difficulty of cultivating species that cannot be propagated outside their natural habitats and that they have to travel long distances for several hours to get the needed medicinal plants. Alternatively, healers may choose to get (on appointed date) such plants upon cash payment for people who are living in the vicinity of the medicinal plants. Medicinal plants that are known to have additional uses (ornamentals, fuel, forage, spice, food and soil conservation) in the area were planted most frequently in homegardens and farmlands. *Allium sativum*, *Foeniculum vulgare*, *Lepidium sativum*, *Ocimum gratissimum*, *Ruta chalepensis*, *Schinus molle* and few others were commonly planted.

Furthermore, the District administration has started considering the indigenous knowledge of the people as testified by the priority given to establish traditional health practitioners association along with the provision of some technical training and discussion on biodiversity conservation concepts. A good justification for the above scenario is the observation during our field study in the area the mutual exchange of knowledge and remedies at the time of monthly meetings. The first author had a chance to participate in two of their meetings and was kindly given permission to record the information.

## Discussion

Despite the efforts made, only few women could take part in the study partly because of the tradition and being the usual case when the interviewers are men as in our case. Women are generally not expected to appear in public or discuss with stranger men both by society and family (husbands deny permission in most cases) or other socio-cultural reasons, which our female informants refrained from describing openly. There were very few women practitioners in the community. More informants are expected to yield more knowledge of plants procured from the wild as was reported by other researchers [33–35]. The rich ethnoecological knowledge was revealed in their elaboration and categorization of the ecological units. They recognized six landscape, five soil and five vegetation types, reflecting their deep understanding of the differences and similarities in these key environmental components. This emanates from the ethnobotanical/ethnoecological knowledge that was shaped over generations and which they use for describing, managing and utilizing the land, the soil and vegetation. Their knowledge also stretches to the individual plants which they grouped into use categories, morphological classes and adaptive forms. Soils which were identified based on colour and texture are applied to determine and select those suitable for the type of crop varieties to be grown on a specific land. This knowledge shares similarities with the modern classification system [36] and the system used in another part of Ethiopia [37]. Such broad-based indigenous knowledge systems are indicative of prolonged experience, relationship and interaction of people with the biotic and abiotic components of the environment as rightly described for other areas in Ethiopia [38–40].

The top three families (Asteraceae, Fabaceae and Solanaceae) reported in this study are among those represented with higher number of taxa in the Ethiopian flora [39–44] and also found to have higher number of medicinal plants by other researchers working in other parts of Ethiopia [45–47]. This might be related to possession of more species that are widely distributed in almost all ecological areas and habitats since the Fabaceae and Asteraceae are respectively the first and third largest families of angiosperms in the Ethiopian flora [48]. These two families have many uses for the community as reported by other researchers [44–46, 49]. The diversity of genera and families (29 with 2–14 species in many genera) is a good indication for the study area being an important reservoir of medicinal plants and ethnomedicinal knowledge. Dependence on a great diversity of plant species for treatment of ailments is a good indicator of profound knowledge on medicinal plants. The six most cited medicinal plants that have relatively higher percentages of informants’ consensus could be considered for further analyses. The fact that *Achyranthes aspera* came both in the most cited and most effective medicinal plants for treating different diseases may indicate that in the long term this species could be locally threatened due to overharvesting. At the time of field data collection, the species was found widely distributed in both the wild lands as well as in and around homegardens.

Eight to fifty-five medicinal plant species recorded in this study have also been documented as medicinal in other parts of Ethiopia as our review of 20 sources [34, 39–44, 46, 49–60] showed. This analysis confirms that those medicinal plants are important in the healthcare systems of different cultures in Ethiopia. On the other hand, 31 of the medicinal plant species reported in our study have not been mention in any of the ethnobotanical literature sources reviewed [34, 39–44, 46, 49–60] suggesting that while the knowledge is shared in some respects it also has some uniqueness to the study communities.

The finding that shrubs and herbs were the most abundant medicinal plants indicated that people rely more on such plants, which may relate to the fact that they are relatively common compared to other growth forms. Other researchers [41, 47, 53, 59, 61] also found that shrubs and herbs are the most frequent medicinal plant species. Most of the wild medicinal plants were accessed from Tara-gedam and Amba forests. Healers and some knowledgeable members of the local community were seen cultivating some medicinal plants in their homegardens for easy access and use of fresh parts at times of remedy preparation. The distribution of medicinal plants in the wild, homegardens and in both premises [39–41, 62, 58] as well as finding of more species in the wild environments were reported by other researchers [33, 43, 47, 62] in Ethiopia and other countries [63, 64].

Use of diverse plant species in the treatment of ailments implied that the people of the study area to date prevent and cure human and livestock ailments with plant materials collected from the surrounding areas. Less number of livestock diseases and medicinal plants were reported compared to those of humans, which could probably be due to the fact that the people give more attention to human ailments compared to livestock diseases. Generally, the local people affirmed that they first try to find medicines for human ailments and then search for remedies for livestock ailments as reported in other areas [38]. The healers also mentioned that they refer to the pharmacopeias (ancient herbals written on parchment) to learn about medicinal plants and treatments for human diseases. Traditional pharmacopeias have also helped to transfer the knowledge to more people. Treatment of human ailments like womb problem, sterility of females, prolonging the life of embryos in the uterus, expelling foreign particles from the eyes and ears, and livestock ailments like increasing sexual needs and beating with stick are new plant uses not encountered in any of the previous publications reviewed.

Higher ICF values as in external parasites, beating with stick and sun stroke in the case of livestock, and febrile illness, headache, anemia, brain tension and malaria in human being are indicative of the presence of similar ethnomedicinal plant knowledge and their continued usage in similar ways among community members [32, 64] as also reported from other parts of Ethiopia [39–41].

*Cordia africana*, the most multipurpose species as in other areas [62], would be imagined to be most threatened in the future. The clue to this is its rare occurrence with sparse distribution around farmlands and some homegardens. This scarcity was due to over harvesting not only for medicinal purpose, but also for other uses, notably for timber production. All of the medicinal plant species and the top ranking ones in particular need urgent conservation actions and adoption of a suitable system of sustainable use.

The preferences of leaves and roots to other plant parts could be attributed to ease of preparation, the presence of medicinally active secondary metabolites and accessibility at the required time in the same manner as described for western [34], southern [45–47, 53, 59–61], northern [41, 58], central [62] and eastern [65] Ethiopia and other countries [63, 64]. The use of leaves for medicinal purposes is less likely to be destructive especially relative to the use of roots. The latter is likely to have negative influence on the survival of the plant. Cultural practices and beliefs requiring digging up of three or seven plants to prepare just a single remedy have been recorded. In some cases three or five or seven pieces each had to be removed from the same or different individual plants and applied to cure the disease, which would likely be unfavorable to conservation. Preparations made from all parts, three and two plant parts for remedy formulations (few in our case) may endanger the species unless mechanisms for sustainable utilization are put in place. Single plant preparations are easier to extract the curative chemical compounds as reported by others [33]. However, mixtures are expected to be more effective due to the additive effects of the combination of plants by increasing the compounds that could act on different pathogens.

Higher frequencies of crushed forms could be related to the ease of preparation at any place, using stones at most, which could be done by most local community members. Informants asserted that medicinal plant parts crushed and soaked in water lead to effective and immediate response to health problems. Crushing came out as the most frequent preparation method in other works [38]. A prescription that required crushed roots of *Asparagus africanus* concocted with honey and stored for seven days in a bottle was used for the treatment of impotency. Healers explained that such a preparation helps to extract the active chemicals and this is analogous to the methods used in modern phytochemical and pharmacological extractions using different solvents in the laboratory. This hints at a fair understanding of the local people about the science behind the traditional practices of herbal remedy preparation and treatment. About 71% of the medicinal plant remedies were prepared from fresh plant material highlighting that live medicinal plants have to be found near homes for instant use. Most herbalists advised that fresh material are more effective for treatment than dried forms further elaborating that drying could easily distort the efficacy of the medicine, and that stored plant medicine is culturally less liked and was also reported by other researchers [41, 53, 59] in Ethiopia. In modern herbal medicine, some secondary metabolites having active healing potentials are known to be quickly transformed to permanent compounds losing their healing power soon upon cutting [5, 8]. The use of dried plants and stored remedies were reported by very few healers, who said that they use dried plant material when availability of fresh material is seasonal. Dependency on fresh material is likely to throw the species to serious threats as had been warned by other sources [39].

Informants affirmed that after the New Year holiday, preparations from the past year could not have the potential to cure ailments if not buried on the eve of the holiday upto the next day to respect the cultural and religious beliefs. The newly prepared remedies are believed to have active constituents such as (volatile oils and other phytochemical and pharmaceutical ingredients) and these could be lost progressively due to factors including temperature, oxidation and reduction. This tradition of collecting most of the medicinal plant materials once in a year has the merit of minimizing overharvesting. Various sources from central [33], western [34], southern [46, 58–60], eastern [62] and northern [41] Ethiopia proclaim that oral route is most frequent. Some sources [33, 34, 58] that recorded measurements for remedies in a similar manner to ours noted the lack of precision and standardization as a drawback of the traditional herbal healthcare system. Additives are included in the medicines to minimize discomfort, improve the taste and reduce adverse effects such as vomiting and diarrhea, and enhance the efficacy and healing potential as explained by the informants. Mixing and using some medicinal plants with common foods and drinks is an easy way for effective treatment, particularly for children and facilitation of ingesting bitter tasting formulations as described in other sources [33, 34, 58].

The recorded taboos and other ritual-like actions related to the collection, preparation and administration of traditional medicine are beliefs carried over generations in the study area in a similar manner to the research results reported from Bale [52] in southeast Ethiopia. The interpretations correspond to healers’ perceptions of medicine and disease treatment whose scientific verification awaits further studies.

Elderly members of the society (aged 51–85 years) had expectedly more knowledge on medicinal plants and their uses due to their long-lasting direct and regular contact with the forests and other plant resources. In contrast, the younger generation is more exposed to modern education and hence not interested in learning and practicing the ethnomedicinal wisdoms, which may affect the continuity of indigenous knowledge. Medicinal plant knowledge difference among age groups was also reported in other studies [2, 45, 59, 66] but one study from southern Ethiopia [47] deviated from this.

People living far away from forests (Asiba and Yifag Akababi) knew relatively fewer species than those residing near the forests (Washa Indiras, Kualla Yihuans and Kidanemhret) showing that contact with the plant resources helps to preserve and continue using the knowledge. Tibabosgie village being close to the forest reported less knowledge due to being more dependent on a few highly knowledgeable healers for their healthcare delivery. Mantogera village is close to Addis Zemen Town and the people have better access to modern medical system than traditional medicine. On the high land area of Aguat Mafsesha, the people live concentrated within a specific compatible area and intensive cultivation is the norm. Here, biodiversity is considerably reduced and the possibility of finding medicinal plants has been minimized.

The study confirmed that variation exists in species preferences among sites, partly due to the wide array of ecological niches within short distances. This is in turn expected to bring about differences in indigenous knowledge among informants of different sites. Similar trends have been reported in a study conducted in eastern central Ethiopia [38]. Though results indicated relative variations between town and rural villages, indigenous medicinal plant knowledge difference was hardly noticeable indicating that even town dwellers living close to forests keep considerable ethnobotanical knowledge as reported in other studies [67, 68].

It is no wonder that agriculture is the main culprit for the loss of medicinal plant habitats, vegetation and species because the communities in the study area depend more on mixed agriculture as their main economic activity with limited landholding and high human population [34, 59, 63, 69]. Low living standards and lack of alternatives are major factors responsible for the decline of forest resources [14]. Cultivating the useful plants in homegardens is crucial, but conservation in the natural wild setting (*in*-*situ*) must also be considered since plants in their natural ecological area can grow at the limits of their potentials and provide the expected results including efficacy as medicine. Sustainable medicinal plant management and conservation are imperative for rural people’s healthcare and community well-being. The importance and conservation purposes of church forests have previously been reported [70]. Likewise, the governmental plant nursery in Addis Zemen Town is used as a germplasm source for the forest as well as the surrounding areas. The nursery is engaged in raising seedlings of selected species that are distributed for reforestation and afforestation programmes, which needs further enhancement and scaling up.

## Conclusion

The present study showed that Tara-gedam and Amba forests harbour a high diversity of medicinally useful plants and the people living in the area have a long history of plant use, and that of medicinal plants is exceptionally notable and culturally rooted in the area. Despite the gradual socio-cultural transformation, the inhabitants have retained remarkable knowledge of the plants and their uses. Difficulties in knowledge transfer and the resulting generation gap in knowledge are threatening the continuity of the medicinal plants and the indigenous knowledge on them. On the other hand, the study provided evidence that medicinal plants will continue to play an important role in the healthcare system in the study area, given support through conservation and education. Knowledge and herbal medical practices for the treatment of various ailments among both rural and urban people are major parts of their livelihoods and culture. The traditional knowledge of the use and conservation of these plants is still being transferred from generation to generation, but appeared to be aging. The problem of transfer of knowledge from the elders to the young generation probably arose following the introduction of modern education, religious, spiritual and culture-related factors. Therefore, it is not only essential to conserve such a wealth of information hidden among the local people but also to apply modern science and technology to meet the ever increasing requirements of humankind. Furthermore, conservation of these biological resources is very important because their sustainable use can generate higher levels of employment and income.
